# KRAS G12V mutation-selective requirement for ACSS2 in colorectal adenoma formation

**DOI:** 10.1016/j.celrep.2025.115444

**Published:** 2025-03-24

**Authors:** Konstantin Budagyan, Alexa C. Cannon, Adam Chatoff, Dorothy Benton, Alison M. Kurimchak, Daniela Araiza-Olivera, Anastasiia Gerasimova, Nathaniel W. Snyder, James S. Duncan, Cristina Uribe-Alvarez, Jonathan Chernoff

**Affiliations:** 1 Department of Biochemistry & Molecular Biology, Drexel University College of Medicine, Philadelphia, PA, USA; 2 Department of Cancer & Cellular Biology, Lewis Katz School of Medicine, Temple University, Philadelphia, PA, USA; 3 Cancer Signaling & Microenvironment Program, Fox Chase Cancer Center, Philadelphia, PA, USA; 4 Lead contact

## Abstract

Oncogenic KRAS mutations are prevalent in colorectal cancer (CRC) and linked to poor prognosis and therapeutic resistance. Emerging evidence suggests that specific KRAS mutations differentially influence treatment responses. In this study, we generate isogenic *Apc*-null mouse colon epithelial cells with four common KRAS mutations. Transcriptomic and proteomic analyses reveal significant enrichment of cholesterol and lipid metabolism pathways in KRAS G12V cells, driven by increased SREBP1 expression and mTORC1 activation. Furthermore, KRAS G12V cells exhibit elevated ACSS2 expression and greater dependence on ACSS2 for proliferative advantage compared to other mutants. Inhibition of ACSS2 uniquely sensitizes KRAS G12V cells to MEK inhibition, highlighting a distinct therapeutic vulnerability. Finally, ACSS2 plays a critical role in early KRAS G12V adenoma development, unlike in KRAS G12D adenomas. These findings highlight mutation-specific metabolic reprogramming in KRAS-driven CRC and identify ACSS2 as a potential therapeutic target.

## In brief

Budagyan et al. demonstrate that different KRAS oncogene mutations drive distinct signaling programs in colon adenoma cells. Specifically, KRAS G12V cells exhibit unique metabolic dependency on ACSS2 and acetate metabolism for proliferative advantage. Targeting ACSS2 selectively sensitizes KRAS G12V cells to downstream inhibition, revealing a therapeutic vulnerability.

## Graphical Abstract

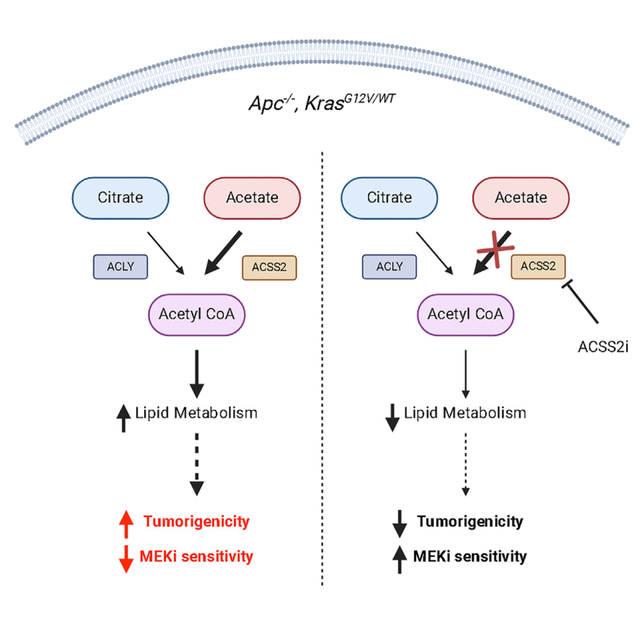

## INTRODUCTION

Colorectal cancer (CRC) is one of the most common malignancies worldwide.^1^ *KRAS* mutations are observed in approximately 45% of CRC cases and are clinically associated with tumor invasion and metastasis, resistance to therapy, and a poor overall survival.^2–4^ The most common sites of oncogenic *KRAS* mutations are codons 12, 13, 61, 117, and 146. Unlike other cancers that are associated with KRAS mutations, CRC is unique for its diversity of *KRAS* alleles, with the most common being G12D (28%), G12V (20%), and G13D (16%).^4,5^ Like other small GTPases, KRAS acts as a binary molecular switch between an active GTP-bound state and an inactive GDP-bound state. Guanine nucleotide exchange factors (GEFs), such as SOS, RASGRF, and RasGRP, assist in the exchange of GDP with GTP.^6–8^ GTPase activating proteins (GAPs) such as NF1 or p120GAP mediate GTP hydrolysis and help inactivate RAS.^9,10^ Activating mutations in *KRAS* result in aberrant Ras signaling due to an altered balance between the active and inactive forms by reducing GTP hydrolysis or by increasing the rate of GTP loading. In this active state, Ras activates many downstream effector pathways such as mitogen-activated protein kinase (MAPK) and phosphatidylinositol 3-kinase (PI3K) -protein kinase B (AKT) signaling, which are responsible for cell-cycle progression, protein synthesis, pro-growth metabolism, and overall cell survival.

Recent studies have shown differences in the biochemical and signaling properties of common KRAS variants.^11–15^ For example, both G12D and G12V mutations have reduced affinity for the Rapidly Accelerated Fibrosarcoma protein (RAF); however, the G12V mutation is predicted to activate RAF better because of its reduced GTPase activity.^12^ Distinct mutant-specific properties suggest that each mutation differently influences the prognosis of the disease and the response to therapy. For example, among the most common KRAS mutations found in CRC, G12V is associated with worse overall survival.^16,17^ Although there is evidence to suggest biological differences between mutant G12V and other *KRAS* allele mutations, these mechanistic distinctions remain unclear.

Recent efforts to target KRAS directly have led to the US Food and Drug Administration (FDA) approval of allele-specific KRAS^G12C^ inhibitors; however, G12C mutations only account for ~3% of CRC cases.^18^ Furthermore, unlike in non-small cell lung cancer (NSCLC), in CRC, the initial response rates to G12C inhibitors are poor.^19,20^ Potential resistance mechanisms include upstream activations of several receptor tyrosine kinases and faster signaling rebound, highlighting the challenges of targeting KRAS-mutant CRC.^21^ The development of other mutant-specific as well as pan-KRAS inhibitors is promising; however, given the limited clinical outcome of G12C inhibitors in CRC, these agents most likely will need to be administered in combination with other drugs. Therefore, a better understanding of the different biological properties of mutant KRAS alleles that contribute to their clinical properties is essential for the development of successful allele-specific therapeutic interventions.

In this study, we explored the signaling differences between KRAS alleles using *Kras* isogenic *Apc*^−/−^ mouse colon epithelial cell lines. We generated isogenic cell lines by altering the original *Kras*^*G12D*^ allele to *Kras*^*G12V*^, *Kras*^*G12R*^, or *Kras*^*G13D*^ using CRISPRdriven genome editing. These cell lines were then used for transcriptomic and proteomic analyses to investigate signaling differences. We focused on the unique properties of KRAS G12V compared to other common KRAS mutations found in CRC. Both analyses indicate significant differences in pathways related to cholesterol and lipid regulation. We found that these processes are upregulated in KRAS G12V lines through increased translocation of the Sterol Regulatory Element Binding Protein 1 (SREBP1) to the nucleus and increased activation of mTORC1 effectors. Furthermore, KRAS G12V cells showed greater acetate utilization, increased expression of acyl-CoA synthetase short-chain family member 2 (ACSS2), and hyperacetylation of regulatory-associated protein of mTOR (Raptor). ACSS2 inhibition preferentially sensitized KRAS G12V cells to mitogen-activated protein kinase kinase (MEK) inhibitors. Finally, we showed that ACSS2 is necessary for the formation of KRAS G12V mutant tumors when engrafted into mice. These observations suggest that ACSS2 plays a crucial role early in the development of KRAS G12V mutant tumors and may further facilitate their proliferation by utilizing acetate as a carbon source for lipogenesis and reducing their sensitivity to MEK inhibitors. Targeting ACSS2 may be a promising therapeutic approach to target KRAS G12V mutant adenomas and/or early-stage CRC.

## RESULTS

### KRAS mutations drive distinct downstream signaling profiles in mouse colon epithelial cells

**Figure 1.**
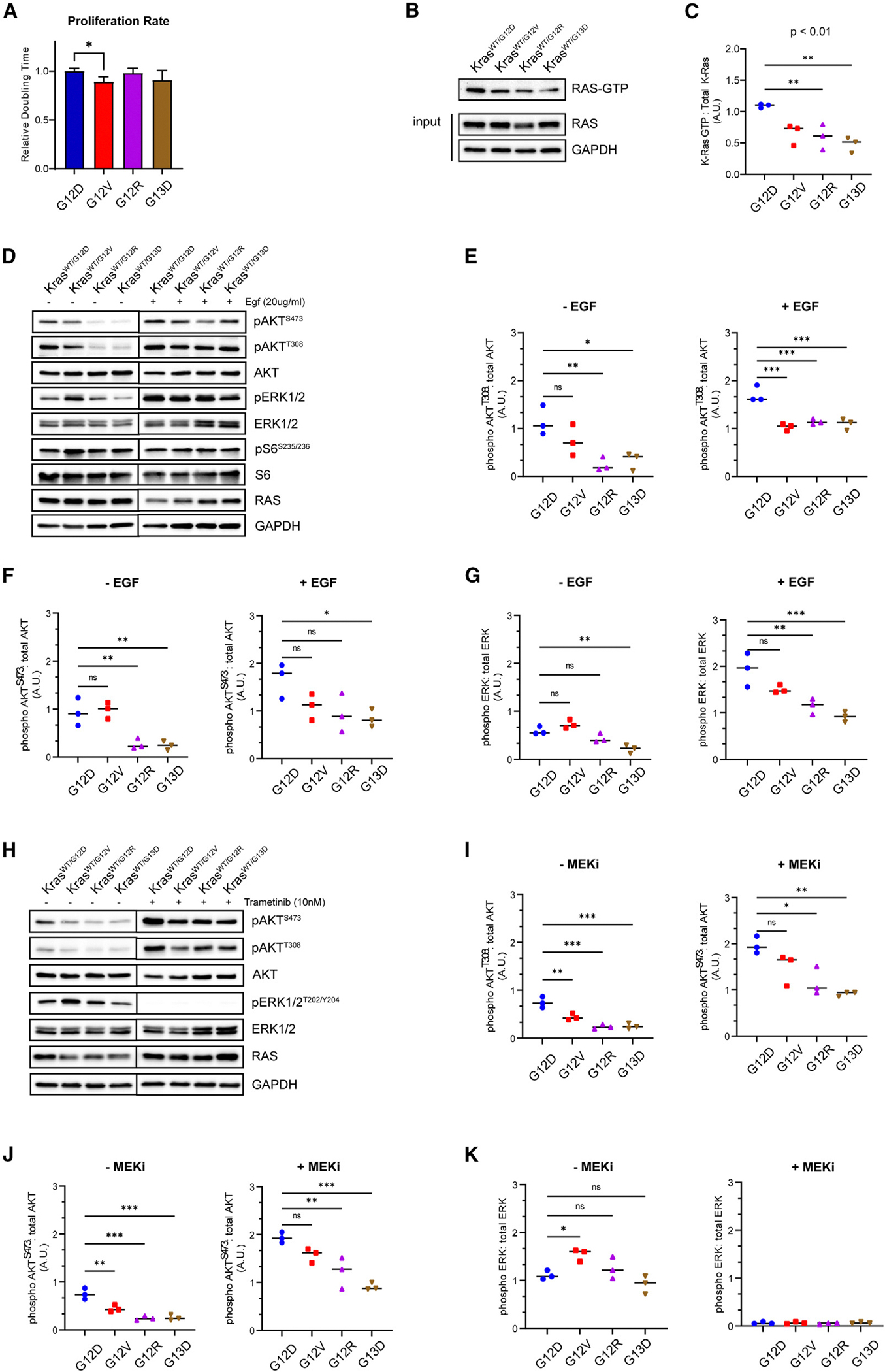
KRAS mutations drive distinct downstream signaling profiles in mouse colon epithelial cells (A) Relative doubling time of KRAS-mutant cells measured by xCELLigence RTCA; *p < 0.05. (B) Representative blot for affinity purification of active Ras using Raf-RBD. Results are representative of three similar experiments. (C) Quantification of band intensities of (B). One-way ANOVA, **p < 0.01. (D) Representative western blot for downstream signaling in KRAS-mutant mouse colon epithelial cells cultured in 0.5% fetal bovine serum (FBS) and upon stimulation with EGF for 20 min. Results are representative of three similar experiments. (E–G) Quantification of western band intensities in (D) for p-Akt^T308^(E), p-Akt^S473^(F), and p-Erk (G). One-way ANOVA; ns, not significant; *p < 0.05, **p < 0.01, ***p < 0.001. (H) Representative western blot for downstream signaling in KRAS-mutant mouse colon epithelial cells in response to 24-h MEK inhibition (10 nM trametinib). Results are representative of three similar experiments. (I–K) Quantification of western band intensities in (H) for p-Akt^T308^(I), p-Akt^S473^(J), and p-Erk (K). One-way ANOVA; ns, not significant; *p < 0.05, **p < 0.01, ***p < 0.001.

The role of mutant *KRAS* alleles on cellular proliferation, downstream signaling, and sensitivity to inhibitors of downstream effectors was evaluated in a mouse colon epithelial isogenic cell-line series.^22^ Proliferation was assessed using the xCELLigence RTCA system, which was used to calculate doubling time during logarithmic growth. Modest differences in proliferation were noted between the cell lines carrying each of the different *Kras*-mutant alleles, with KRAS G12V cells showing a shorter doubling time (Figure 1A).

Next, we investigated the allelic influence of KRAS activation in mouse colon epithelial cells by immuno-precipitation with the RAS-binding domain of RAF1 (Raf-RBD). Our results showed that KRAS G12D cells had the highest level of GTP-bound KRAS, indicating a higher level of KRAS activation compared to the other mutants. KRAS G12V cells had a slightly lower level of active RAS; however, the difference was not statistically significant. KRAS G12R and G13D had significantly lower levels of GTP-bound KRAS than KRAS G12D (Figures 1B and 1C).

To assess the allelic consequences on downstream signaling, we evaluated the activation of the PI3K and RAF pathways.^23,24^ Under low-serum culture conditions, there was no difference in PI3K activation between KRAS G12D and G12V, whereas G12R and G13D cells showed significantly decreased level of AKT phosphorylation (Figures 1D–1F). KRAS G13D cells showed lower levels of extracellular signal-regulated kinase (ERK) activation compared to the other cell lines under lower-serum conditions. All KRAS mutants remained responsive to upstream mitogenic signals, as acute EGF stimulation increased AKT and ERK phosphorylation (Figures 1D–1G).

**Figure 2.**
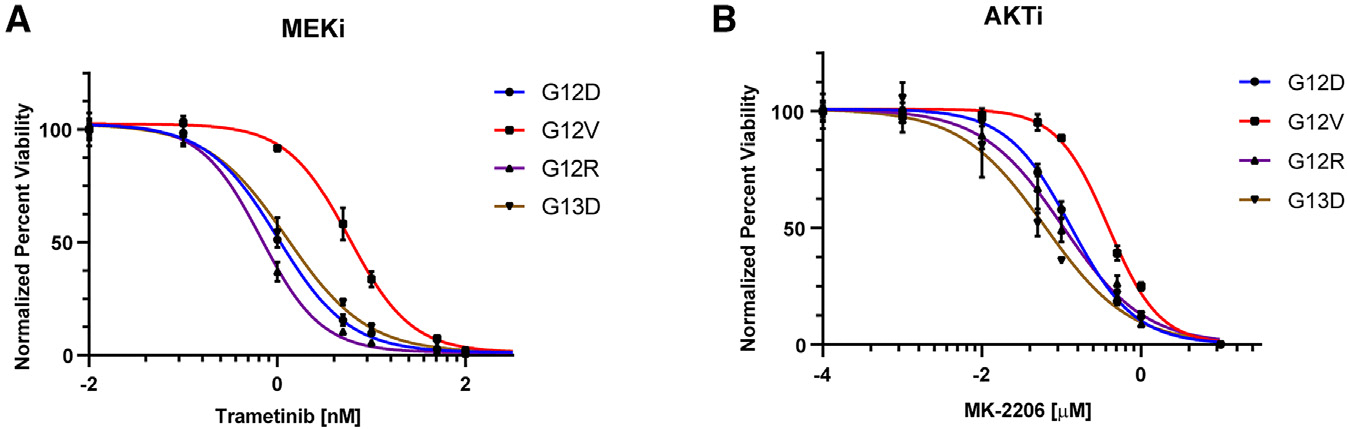
KRAS G12V mouse colon epithelial cells exhibit decreased sensitivity to downstream effector pathway inhibitors Response of KRAS-mutant mouse colon epithelial cells to MEK inhibition (trametinib) (A) and AKT inhibition (MK2206) (B). Cells were cultured at various doses of inhibitor for 72 h. Viability was evaluated using Cell-Titer Glo luminescence. Results from three biological experiments per genotype are shown; curves were fitted with nonlinear regression in GraphPad Prism 9.

In response to a MEK inhibitor, all KRAS mutants responded with increased AKT phosphorylation (Figures 1H–1K), consistent with previous studies highlighting the PI3K/AKT pathway as a major mechanism of resistance to MEK inhibition.^25,26^ To further assess the dependency on downstream effectors, KRAS-mutant cells were evaluated for their sensitivity to selective AKT and MEK inhibitors. Interestingly, the sensitivity profiles were different among the mutants, with G12V cells showing the lowest sensitivity to both inhibitors (Figures 2 and S2).

### Proteogenomic analyses reveal increased mTORC1 activity in KRAS G12V cells

To gain a deeper analysis of allele-specific signaling, we performed global transcriptomics using RNA sequencing (RNA-seq) on multiple independent clones of KRAS-mutant mouse colon epithelial cells. RNA extraction and quality control were performed in synchrony on all samples followed by library preparation and mRNA-seq at the Next Generation Sequencing Facility at the Fox Chase Cancer Center. Principal-component analysis (PCA) of the data revealed that allele replicates clustered together but different genotypes separated in the principal-component space. Samples expressing KRAS G12R clustered closer to those expressing KRAS G13D than to those expressing KRAS G12D or G12V. KRAS G12D and G12V formed separate clusters, indicating a clear distinction between the gene expression profiles of the two mutants (Figure S3A).

**Figure 3.**
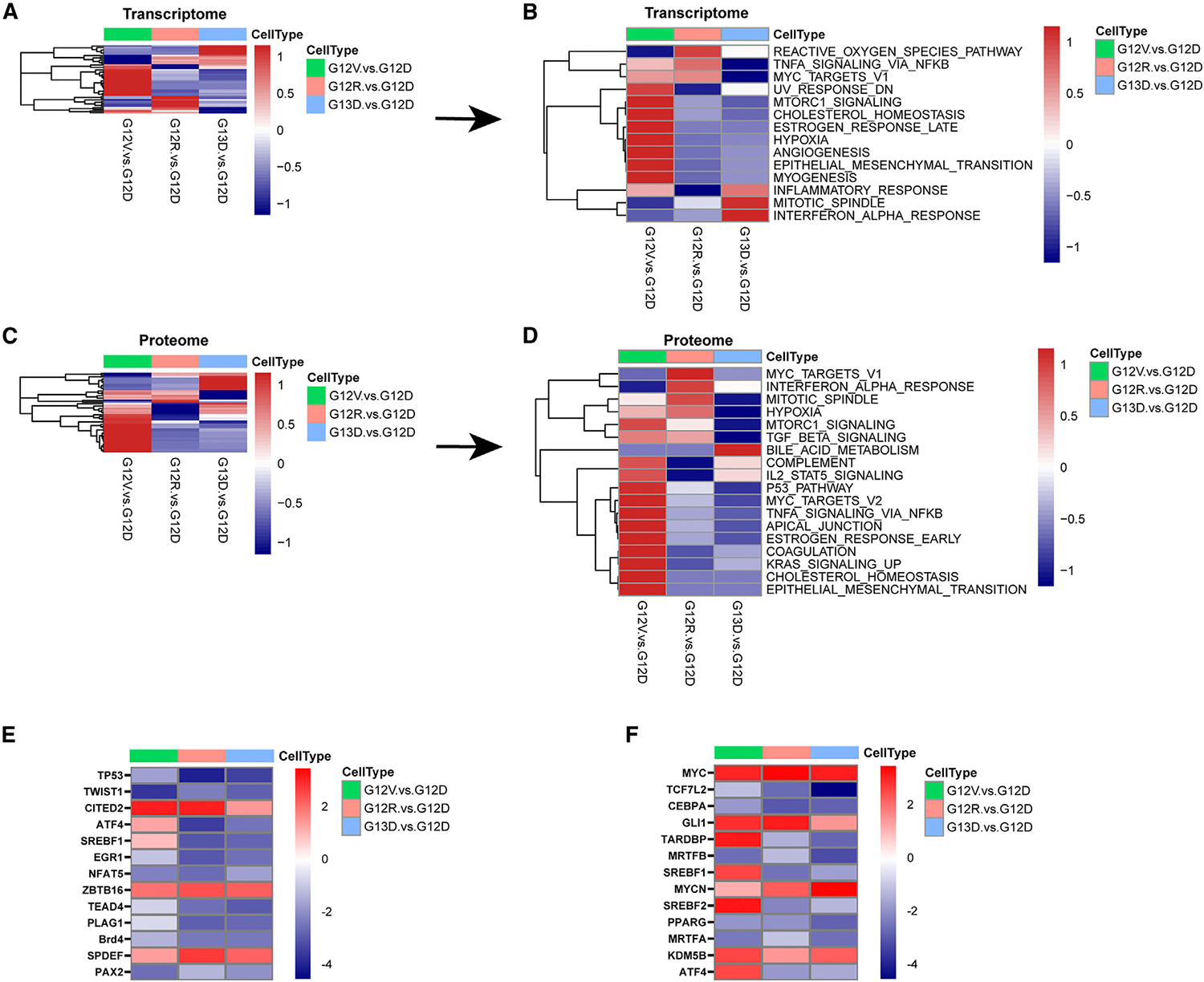
Proteogenomic analyses reveal increased mTORC1 activity in KRAS G12V-expressing cells (A and B) GSEA results for hallmark gene sets, as determined by bulk RNA-seq of KRAS-mutant murine CRC. (A) Global view of GSEA results comparing G12V:G12D (denoted by green columns), G12R:G12D (denoted by peach color), and G13D:G12D (denoted by blue columns). (B) Detailed heatmap of significantly enriched hallmark gene sets in G12V, G12R, or G13D cells relative to G12D. FDR < 0.05, log fold change (logFC) >2. (C and D) GSEA results for hallmark gene sets, as determined by global proteomic analysis of KRAS-mutant cells. (C) Global view of GSEA results for hallmark gene sets comparing G12V:G12D, G12R:G12D, and G13D:G12D. (D) Detailed heatmap of significantly enriched hallmark gene sets in G12V, G12R, or G13D cells relative to G12D. FDR < 0.05, logFC > 2. (E) Heatmap showing IPA of upstream regulators, as determined by bulk RNA-seq, comparing G12V:G12D, G12R:G12D, and G13D:G12D. Upstream regulators are ranked according to the *Z* score, which predicts activation (red)/suppression (blue). (F) Heatmap showing IPA of upstream regulators, as determined by global proteome analysis, comparing G12V:G12D, G12R:G12D, and G13D:G12D. Upstream regulators are ranked according to the *Z* score, which predicts activation (red)/suppression (blue). FDR < 0.2, logFC > 1.5.

To gain a comprehensive understanding of the RNA-seq data at the pathway level, we conducted a gene set enrichment analysis (GSEA) to identify hallmark gene sets enriched in each mutant compared to the KRAS G12D baseline cell line. Several pathways were significantly enriched or de-enriched in KRAS G12V, G12R, and G13D relative to KRAS G12D (Figure 3A). Given the distinct characteristics of the KRAS G12V mutation, we focused on pathways that were upregulated or downregulated in those mutants. Among these, the MTORC1_SIGNALING and CHOLESTEROL_HOMEOSTASIS gene sets were uniquely enriched. (Figure 3B).

Next, we conducted global proteomic analysis to validate our transcriptomic data. As in our RNA-seq analysis, the proteomic MTORC1_SIGNALING and CHOLESTEROL_HOMEOSTASIS gene sets were consistently enriched in KRAS G12V cells (Figures 3C, 3D, S3D, and S3E). These expression data were similar between multiple independently derived clones (Figures S3B and S3C). To evaluate the upstream transcriptional regulators responsible for the observed gene expression changes, we performed an ingenuity pathway analysis (IPA) of upstream regulators using both transcriptomic and global proteomic data. IPA identified SREBF1, SREBF2, and activating transcription factor 4 (ATF4) as uniquely activated potential upstream regulators in KRAS G12V cells (Figures 3E and 3F). These transcription factors have previously been shown to be regulated by mTORC1.^23,27–29^ These findings reveal distinct signaling profiles for each mutant *Kras* allele, demonstrating their functional differences. Notably, KRAS G12V-expressing cells exhibit elevated mTORC1 activity, driving signatures associated with enhanced lipogenesis.

### KRAS G12V cells show increased acetate utilization through ACSS2

**Figure 4.**
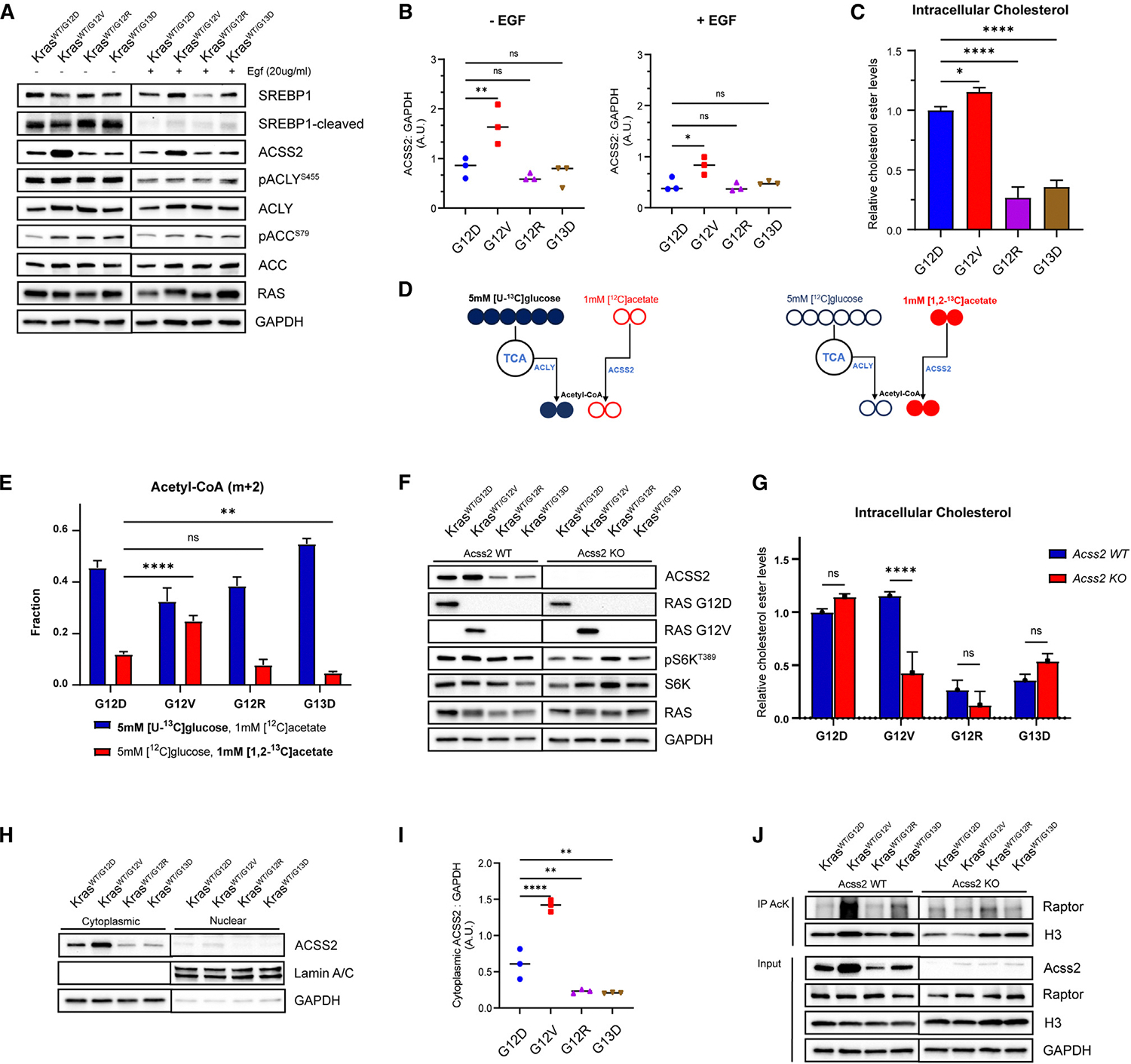
KRAS G12V cells show increased acetate utilization via ACSS2 (A) Representative western blot for expression and activation of lipogenic enzymes in mouse colon epithelial cells cultured in 0.5% FBS and upon stimulation with EGF for 20 min. Results are representative of three similar experiments. (B) Quantification of western band intensities in (A) for ACSS2. One-way ANOVA; ns, not significant; *p < 0.05, **p < 0.01. (C) Intracellular cholesterol ester levels in KRAS-mutant cells. Data show Cholesterol Ester-Glo luminescence averages from three biological replicates. One-way ANOVA; ns, not significant; *p < 0.05, ****p < 0.0001. (D) Experimental design for heavy isotope labeling of acetyl-CoA using [U-^13^C] glucose with unlabeled acetate (left) and [1,2-^13^C] acetate with unlabeled glucose (right). (E) m + 2 acetyl-CoA following 24-h labeling with 5 mM [U-^13^C] glucose with 1 mM unlabeled acetate (left) and 1 mM [1,2-^13^C] acetate with 5 mM unlabeled glucose; **p < 0.01, ****p < 0.0001. (F) Representative western blot confirming ACSS2 KO using CRISPR. (G) Intracellular cholesterol ester levels in KRAS-mutant; ACSS2 KO cells. Data show Cholesterol Ester-Glo luminescence averages from three biological replicates. One-way ANOVA; ns, not significant; ****p < 0.0001. (H) Representative western blot of ACSS2 subcellular localization in KRAS-mutant cells. (I) Quantification of western band intensities in (H) for cytoplasmic ACSS2. One-way ANOVA; **p < 0.01, ****p < 0.0001. (J) ACSS2-dependent acetylation of RAPTOR in KRAS G12V CRC cells. Proteins were immunoprecipitated with anti-acetylated lysine antibodies (Abs), and the immunoprecipitates (IPs) were probed with anti-RAPTOR Abs.

Given the strong mTORC1 and cholesterol biosynthesis signature observed in KRAS G12V cells, we explored these pathways further. One of the major mechanisms of mTORC1-driven lipogenesis is the regulation of transcription factors and sterol regulatory element-binding proteins (SREBPs). Oncogenic and growth factor signaling in cancer cells increases mTORC1 signaling, resulting in the cleavage and translocation of SREBPs from the endoplasmic reticulum to the nucleus, where they upregulate genes for *de novo* lipid and cholesterol synthesis.^30^ SREBPs play a crucial role in regulating genes encoding ATP-citrate lyase (ACLY), ACSS2, and acetyl-CoA carboxylase (ACC), among others that drive lipogenesis.^27,28,31^ Western blot analysis was performed to evaluate the influence of KRAS mutations on the expression of key proteins driving lipogenesis. Under low-serum culture conditions, all four mutants exhibited increased levels of cleaved SREBP1, indicating an increase in the SREBP1 nuclear fraction that drives lipogenesis (Figures 4A, S4A, and S4B).

Cytoplasmic acetyl-coenzyme A (acetyl-CoA) is a central metabolic intermediate that acts as a precursor for lipogenesis. When acetyl-CoA is needed, citrate is cleaved by the ACLY to generate oxaloacetate and acetyl-CoA.^32^ In tumor cells, an alternative route converts cytoplasmic acetate to acetyl-CoA using ACSS2.^33,34^ Western blot analysis revealed no significant differences in the phosphorylation of ACLY; however, the expression of ACSS2 was significantly elevated in KRAS G12V cells under low-serum conditions and remained elevated after growth factor stimulation (Figures 4A, 4B, and S4A). These findings suggest that ACSS2 may be a crucial contributor to the elevated lipogenesis observed in KRAS G12V mutant cells. To confirm the elevated expression of *Acss2* in KRAS G12V mutants, qPCR analysis was performed by comparing the expression levels relative to KRAS G12D cells. Different KRAS G12V clones consistently showed a >2-fold increase in *Acss2* expression (Figure S4C).

To investigate the relationship between ACSS2 expression and intracellular cholesterol levels, we measured cholesterol levels in KRAS-mutant mouse colon epithelial cells. KRAS G12V cells exhibited the highest intracellular cholesterol levels compared to other mutants (Figure 4C). Interestingly, KRAS G12V cells did not exhibit increased sensitivity to inhibition of HMG-CoA reductase, the rate-limiting step of cholesterol synthesis, compared to KRAS G12D cells, suggesting that there are additional pathways that contribute to the decreased sensitivity of KRAS G12V cells to targeted inhibitors (Figure S4D).

Elevated levels of ACSS2 in KRAS G12V cells suggest an increased utilization of acetate to generate cytoplasmic acetyl-CoA for lipogenesis. To determine the use of glucose- and acetate-derived carbon for cytoplasmic acetyl-CoA generation, we performed stable isotope tracer experiments. KRAS-mutant cells were incubated with 5 mM [U-^13^C] glucose and 1 mM unlabeled acetate, or in 1 mM [1,2-^13^C] acetate and 5 mM unlabeled glucose for 24 h (Figure 4D). These experiments showed that KRAS G12D cells received approximately 12% of their acetyl-CoA acyl carbons from acetate, KRAS G12R cells received approximately 10% from acetate, and KRAS G13D cells received approximately 5% from acetate. Interestingly, KRAS G12V cells received about 25% of their acetyl-CoA acyl carbons from exogenous acetate, which was significantly higher than that of other mutants, indicating greater utilization of acetate under normal culture conditions (Figure 4E). Furthermore, we accessed the incorporation of labeled glucose or acetate into 3-hydroxy-3-methylglutaryl coenzyme A (HMG-CoA). Consistently, KRAS G12V-expressing cells exhibited greater incorporation of labeled acetate into HMG-CoA and an overall higher intracellular abundance of HMG-CoA (Figures S4E and S4F).

To further investigate the importance of ACSS2 in KRAS-mutant mouse colon epithelial cells, we generated ACSS2 knockout (KO) cells using CRISPR-mediated gene KO (Figure 4F). Intracellular cholesterol levels in KRAS G12V; ACSS2 KO cells are significantly decreased relative to KRAS G12V; ACSS2 WT. Cholesterol levels in other KRAS-mutant cells were not significantly impacted by the knockdown of ACSS2 (Figure 4G). Altogether, these findings suggest that KRAS G12V cells have greater expression of ACSS2, leading to greater utilization of acetate as a carbon source at normal conditions compared to other mutants, and that ACSS2 KO uniquely affects KRAS G12V mutants, compromising their lipogenesis as indicated by decreased intracellular cholesterol.

### ACSS2-dependent hyperacetylation of RAPTOR in KRAS G12V cells

ACSS2 is present in the nucleus and the cytoplasm of cells. While cytosolic ACSS2 plays a critical role in the *de novo* biosynthesis of lipids, nuclear ACSS2 is involved in the recycling of acetate produced by histone deacetylation reactions.^33–35^ Subcellular localization of ACSS2 in KRAS-mutant mouse colon epithelial cells was analyzed, indicating that ACSS2 was primarily localized in the cytoplasmic fraction. Consistently, KRAS G12V mutants exhibit higher expression levels relative to other mutations (Figures 4H and 4I), suggesting a greater impact on lipid metabolism.

In addition to its role in lipid metabolism, ACSS2 is also involved in the regulation of histone and protein acetylation.^36^ Given the cytoplasmic localization of ACSS2 in the mouse colon epithelial cells, we considered potential cytosolic targets of acetylation. Recent studies demonstrated that RAPTOR, a key component of mTORC1, is activated by the acetylation of K1097.^35^ Intriguingly, we found that RAPTOR is heavily acetylated in G12V cells and that this acetylation is dependent on ACSS2 (Figure 4J). This observation suggests a potential link between ACSS2, mTORC1, and lipid synthesis in G12V cells; however, further studies are needed.

### ACSS2 inhibition sensitizes KRAS G12V mouse colon epithelial cells to MEK inhibition

**Figure 5.**
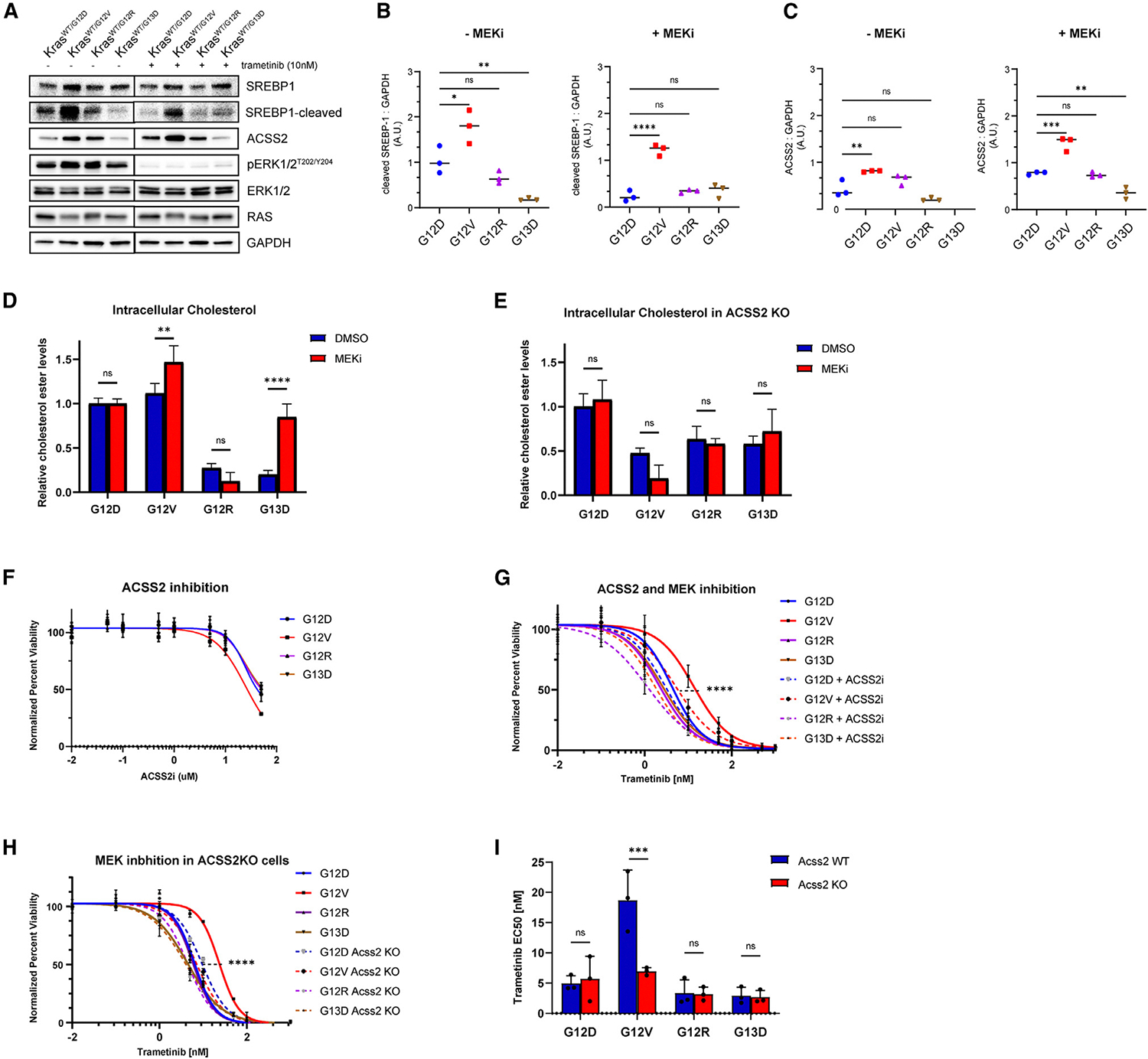
ACSS2 inhibition sensitizes KRAS G12V mouse colon epithelial cells to MEK inhibition (A) Representative western blot for SREBP-1 and ACSS2 in mouse colon epithelial cells in response to 24-h MEK inhibition (10 nM trametinib). Results are representative of three similar experiments. (B and C) Quantification of western band intensities in (A) for cleaved SREBP-1 (B) and ACSS2 (C). One-way ANOVA; ns, not significant; *p < 0.05, **p < 0.01, ***p < 0.001, ****p < 0.0001. (D) Intracellular cholesterol ester levels in KRAS-mutant cells in response to 24-h MEK inhibition. Data show Cholesterol Ester-Glo luminescence averages from three replicates. One-way ANOVA; ns, not significant; **p < 0.01, ****p < 0.0001. (E) Intracellular cholesterol ester levels in KRAS mutant; Acss2 KO mouse colon epithelial cells in response to 24-h MEK inhibition. Data show Cholesterol Ester-Glo luminescence averages from three replicates. One-way ANOVA; ns, not significant. (F) Sensitivity of KRAS-mutant mouse colon epithelial cells to ACSS2 inhibition. Data show Cell-Titer Glo luminescence averages, curves were fitted with nonlinear regression in GraphPad Prism 9, and extra sum of squares f test was used to compare difference in the logarithmic value of the drug concentration that produces 50% growth inhibition (logIC50). ****p < 0.0001. (G) Sensitivity of KRAS-mutant mouse colon epithelial cells to combined MEK and ACSS2 inhibition. Data show Cell-Titer Glo luminescence averages, curves were fitted with nonlinear regression in GraphPad Prism 9, and extra sum of squares f test was used to compare difference in logIC50. ****p < 0.0001. (H) Sensitivity of KRAS-mutant, Acss2 KO mouse colon epithelial cells to MEK inhibition. Data show Cell-Titer Glo luminescence averages, curves were fitted with nonlinear regression in GraphPad Prism 9, and extra sum of squares f test was used to compare difference in logIC50. ****p < 0.0001. (I) EC50 values of trametinib sensitivity for each biological replicate, as calculated in GraphPad Prism, were plotted by mutant and ACSS2 status. One-way ANOVA; ns, not significant; ***p < 0.001.

To evaluate the effect of MEK inhibition on SREBP1 and ACSS2 expression, we performed immunoblot in response to trametinib. KRAS G12V cells exhibited higher levels of cleaved SREBP1 compared to other mutants, consistent with proteogenomic data (Figures 5A and S4B). In response to MEK inhibition, levels of cleaved SREBP1 decreased in KRAS G12D, G12R, and G13D mutant cells but remained elevated in KRAS G12V cells (Figures 5A and 5B). Furthermore, KRAS G12D and G12V cells showed increased expression of ACSS2 in response to MEK inhibition, with KRAS G12V cells maintaining the highest level compared to other mutants (Figures 5A and 5C).

Next, we investigated the relationship between intracellular cholesterol levels and ACSS2 expression in response to MEK inhibition. KRAS G12V cells exhibited a significant increase in cholesterol levels following MEK inhibition, while KRAS G12D and G12R cells showed no significant difference. In ACSS2 KO cells, there were no significant changes in intracellular cholesterol levels after trametinib treatment (Figures 5D and 5E). These results suggest that KRAS G12V cells display a unique ability to utilize ACSS2 in response to MEK inhibition for proliferative advantage and may confer greater dependence on the ACSS2 pathway compared to other mutants.

To evaluate the potential dependence of KRAS-mutant cells on ACSS2, a small-molecule inhibitor of ACSS2 was used to evaluate viability. Although there was no significant difference in the sensitivity to ACSS2 inhibitor alone, it increased the sensitivity of KRAS G12V cells to MEK inhibition in a synergistic manner, whereas the sensitivity of other KRAS mutants was not altered (Figures 5F, 5G, S6A, and S6B). Furthermore, genetic knockdown experiments showed consistent results where KRAS G12V; Acss2 KO cells had a significant decrease in sensitivity to trametinib compared to KRAS G12V; Acss2 WT cells; however ACSS2 KO did not alter the sensitivity of other mutants (Figures 5H and 5I). Overexpression of ACSS2 reduced the sensitivity of KRAS G12D and G12V cells to trametinib, with the most pronounced effect observed in KRAS G12V cells. This further supports the role of ACSS2 in mediating resistance to MEK inhibition (Figures S5A and S5B). Given that physiological circulating levels of acetate are higher (50–200 μM) than typically used in media, we repeated the viability assay supplemented with 100 μM acetate, confirming that results were not due to a low-acetate environment (Figures S5C and S5D). ACLY inhibition did not alter the sensitivity of KRAS G12V cells to MEK inhibition, further implying that KRAS G12V cells are reliant on acetate and ACSS2 for enhanced survival (Figures S5E and S5F). Interestingly, addition of exogenous cholesterol did not alter the sensitivity of KRAS G12V cells to MEK inhibition, suggesting that ACSS2’s role in resistance to MEK inhibition is not solely due to elevated cholesterol levels (Figures S5G and S5H). Additionally, we noted that MEK inhibition increased the sensitivity of KRAS G12V cells to rapamycin, an MTORC1 inhibitor; however, ACSS2 inhibition did not (Figures S6C–S6E). Taken together, these data suggest that KRAS G12V mouse colon epithelial cells are dependent on ACSS2 for enhanced survival, which affects their sensitivity to MEK inhibition, rendering them particularly sensitive to ACSS2 inhibition in combination with trametinib.

### Dual inhibition of ACSS2 and MEK in human isogenic cell lines selectively targets KRAS G12V cells

Our results suggest the potential utility of targeting ACSS2 in KRAS G12V colorectal adenocarcinoma. To determine whether the same dependency is evident in human KRAS-mutant CRC cell lines, we evaluated this therapeutic approach in two isogenic human cell line systems (LIM1215 and SW48) that comprise a series of *KRAS*-mutant alleles.

**Figure 6.**
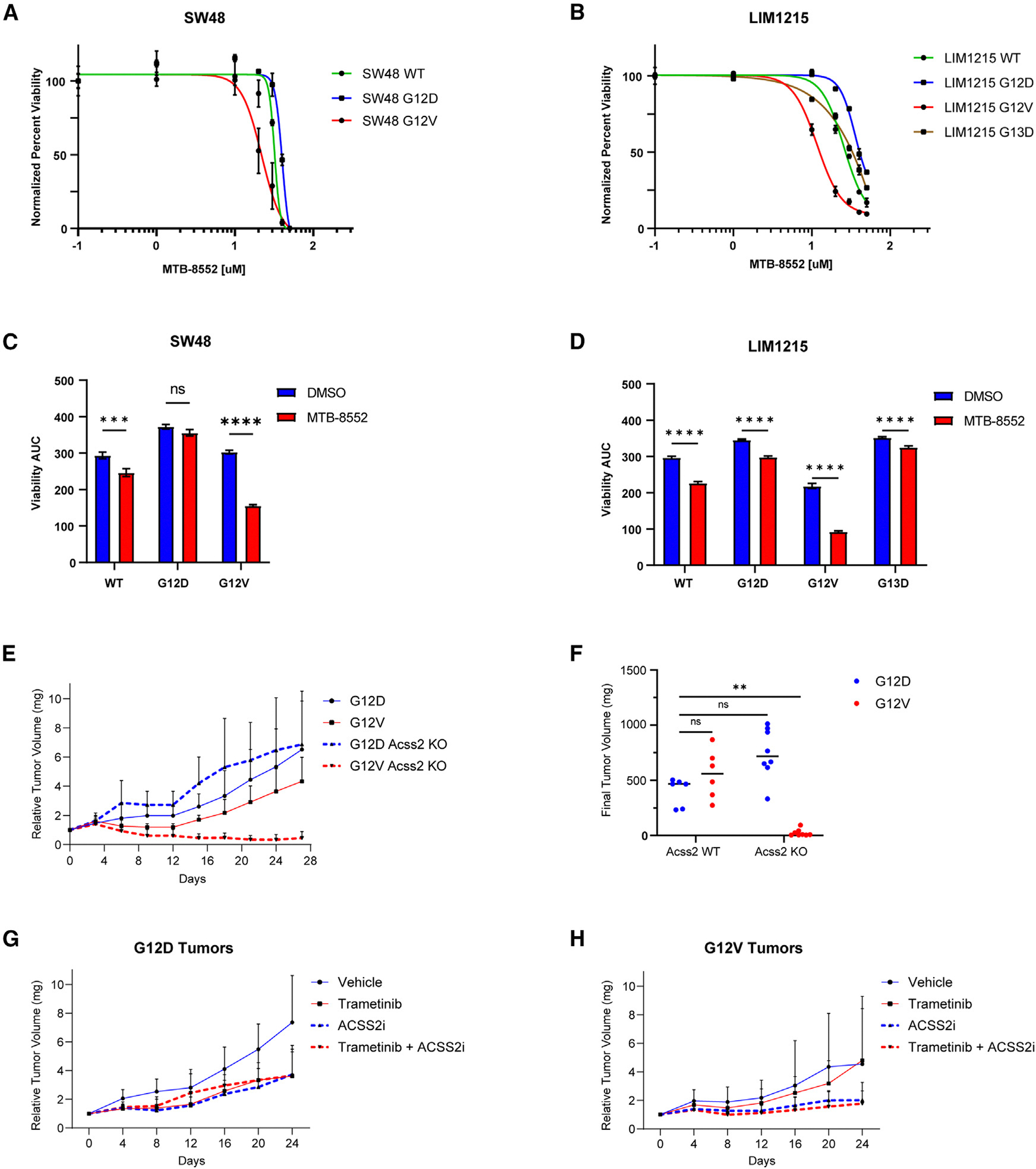
ACSS2 is required for KRAS G12V-driven tumor growth (A) Sensitivity of SW48 KRAS WT, G12D, and G12V isogenic cell line system to an ACSS2 inhibitor. (B) Sensitivity of LIM1215 KRAS WT, G12D, G12V, and G13D isogenic cell lines to an ACSS2 inhibitor. (C) Sensitivity of SW48 isogenic cell lines to MEK inhibition alone or in combination with an ACSS2 inhibitor. Area under the curve (AUC) was calculated in GraphPad Prism. Blue is calculated AUC in response to MEK inhibition alone; red is AUC in response to MEK and ACSS2 inhibition; ***p < 0.001, ****p < 0.0001. (D) Sensitivity of LIM1215 isogenic cell lines to MEK inhibition alone and in combination with an ACSS2 inhibitor. AUC was calculated in GraphPad Prism. Blue is calculated AUC in response to MEK inhibition alone; red is AUC in response to MEK and ACSS2 inhibition; ****p < 0.0001. (E) Relative tumor volume for mice that were allografted with KRAS G12D, KRAS G12V, KRAS G12D; ACSS2 KO, and KRAS G12V; ACSS2 KO cells. Tumor volumes were measured every 3 days for 28 days. (F) Final tumor weight at the end of the experiment. Student t test; ns, not significant; **p < 0.01. (G and H) Relative tumor volume for mice that were allografted with (G) KRAS G12D and (H) KRAS G12V cells. Mice were dosed with 0.2 mg/kg trametinib daily by mouth (p.o.) and/or 15 mg/kg ACSS2i every 2 days intraperitoneally (i.p.).

Unlike mouse colon epithelial cell lines, ACSS2 expression was not significantly different between KRAS mutants, nor was it significantly elevated in response to MEK inhibition in human isogenic CRC cell lines (data not shown). However, as with mouse KRAS cells, both LIM1215 KRAS G12V and SW48 KRAS G12V cells exhibited higher levels of total cholesterol along with a significant increase in intracellular cholesterol in response to MEK inhibition compared to KRAS WT and G12D cells (Figures S7A–S7D). Furthermore, the KRAS G12V cells were more sensitive to ACSS2 inhibition and showed greater sensitivity to ACSS2 inhibition when used in combination with a MEK inhibitor (Figures 6A and 6B). Similar results were obtained in the SW48 isogenic cell line system, suggesting a general sensitivity that could be exploited therapeutically (Figures 6C, 6D, S7E, and S7F).

### ACSS2 is required for KRAS G12V-driver tumor growth

The mouse colon epithelial KRAS-mutant isogenic cell line system replicates the early stage of the “classic” adenoma-carcinoma sequence, which is responsible for approximately 80% of CRC cases. Our findings using this model system suggest that KRAS G12V-expressing cells have higher expression of ACSS2 and are particularly dependent on ACSS2 for their survival. To investigate this hypothesis, we grafted KRAS G12D; ACSS2 WT, KRAS G12V; ACSS2 WT, KRAS G12D; ACSS2 KO, and KRAS G12D; ACSS2 KO cells into the flank of C57BL/6 and evaluated their ability to form tumors. Remarkably, KRAS G12V; ACSS2 KO cells were not able to proliferate despite initial tumor formation (Figures 6E and 6F). These data suggest that ACSS2 plays a crucial role in the early development of KRAS G12V mutant tumors.

Next, we grafted KRAS G12D and KRAS G12V onto the flank of C57BL/6 mice and treated them with vehicle, trametinib, ACSS2 inhibitor, or a combination of trametinib and ACSS2 inhibitor. KRAS G12D tumor growth was significantly inhibited by trametinib and by the ACSS2 inhibitor, although the combination of the two did not add any benefit (Figures 6G and 6H). In contrast, the KRAS G12V tumors did not respond to trametinib alone. However, ACSS2 inhibition significantly inhibited tumor growth, and the addition of trametinib to ACSS2 inhibition further arrested tumor progression.

## DISCUSSION

Decades of research on targeting KRAS in cancer have finally removed the “undruggable” tag from the mutant KRAS. The availability of new KRAS-targeting therapeutic agents has been accompanied by increased appreciation that not all KRAS mutations are created equal. Understanding the biological differences between various mutant alleles can aid in the development of more effective therapeutic strategies for targeting KRAS. In this study, we established that ACSS2 is a unique metabolic regulator in KRAS G12V mutant cells. Our findings further contribute to growing evidence that not all KRAS mutants elicit the same biological effects and will benefit from the same therapeutic regimen.

In this study, we evaluated signaling and functional differences caused by common KRAS mutations using an isogenic mouse colon epithelial cell line model. Through proteogenomic analyses, we demonstrated that KRAS G12V cells exhibit higher levels of cytoplasmic ACSS2 than other mutants. Prior studies have shown that acetate can be used to generate nuclear and cytoplasmic acetyl-CoA, a process that is ACSS2 mediated, which is an essential metabolic precursor for lipogenesis and protein acetylation.^37–39^ In this study, we found that KRAS G12V mutants exhibited greater acetate incorporation into cytoplasmic acetyl-CoAs, and that *Acss2* KO significantly affected the ability of KRAS G12V mutants to generate cholesterol esters but had no effect on other mutants. In addition to increased lipogenesis, these effects may also be due to ACSS2-mediated hyperacetylation of RAPTOR in KRAS G12V cells, activating mTORC1^35^ (Figure 4J).

Acetate is one of the three major short-chain fatty acids (SCFAs) produced by microbial fermentation in the gut and is therefore abundantly available in the colon and rectum.^36^ Although normal acetate levels are associated with a protective function in the colon epithelium, elevated acetate levels have been associated with CRC in humans.^40–42^ Given the findings of this study, the microbial environment in the colon may provide favorable conditions for KRAS G12V adenoma development. Previous studies have shown that cancer cells under stressful conditions, such as hypoxia, can adapt metabolically by increasing acetate utilization via ACSS2 to support proliferation and survival.^43^ The healthy mucosa of the large intestine is already under physiological hypoxic conditions and, as the tumors form and grow, the hypoxic environment is only further exacerbated.^44,45^ Therefore, increased ACSS2 expression in KRAS G12V cells and their inherent increased utilization of acetate may favor KRAS G12V adenoma formation and continuous growth in a hypoxic colorectal environment. These data suggest that targeting ACSS2 early in KRAS G12V-driven adenoma formation could inhibit its ability to proliferate and may be a viable therapeutic option as part of an early combination-therapy intervention.

Under basal conditions, cells primarily catabolize glucose into acetyl-CoA via ACLY, making acetate a secondary carbon source. Therefore, the inhibition of ACSS2 should have minimal effect on normal cycling cells, thus making it an attractive target that can be combined with other treatments. In the present study, we found that ACSS2 expression increases in response to MEK inhibition and contributes to therapy resistance. Given the inherent ability of KRAS G12V cells to utilize acetate via ACSS2, it is possible that these mutants can also utilize ACSS2 in response to other targeted inhibitors. Therefore, ACSS2 inhibition could be further explored in combination with therapeutically relevant agents, including direct pan-KRAS inhibitors currently in clinical trials.

Despite the worldwide prevalence of CRC, clinical success in the treatment of KRAS-driven CRC has been limited. While there have been recent breakthroughs in targeting KRAS G12C in NSCLC and KRAS G12D in pancreatic cancer, there is yet to be a KRAS G12V-specific compound, and there is an unmet clinical need for strategies to target this tumor subtype. These data indicate that ACSS2 is a potential vulnerability in KRAS G12V-mutant CRC. Taken together, this study furthers our understanding of the differences between KRAS mutations and has established the groundwork for future allele-specific anti-RAS therapies.

### Limitations of the study

There are several potential limitations to our studies. First, genetic and/or epigenetic drift might account for some of the observed metabolic differences between the isogenic KRAS-mutant cell lines. We tried to compensate for such changes by studying multiple independently edited clones when possible. In terms of RNA and protein expression, these independent clones showed similar, although not identical, profiles within each type of KRAS-mutant (Figures S3 and S4C) and consistent responses to metabolic inhibitors (Figures S2 and S4D). Second, given that the addition of exogenous cholesterol did not alter the sensitivity of G12V cells to MEK inhibitors (Figures S5E and S5F), the effects of ACSS2 are likely to extend beyond cholesterol synthesis, perhaps involving other lipids or protein modifications. Third, KRAS-mutant cells were grafted into mice as subdermal flank tumors, which differ from the physiological conditions of the colon. An alternative approach is to inject the tumors orthotopically.^46,47^ This would allow for evaluation of the effect of ACSS2 in KRAS-mutant cells under physiological concentrations of acetate in the gut. In addition, these ideas could be tested in a genetically engineered mice model (e.g., mice bearing *Apc*^*fl/fl*^; *Kras*^*Mut/+*^; *Acss2*^−/−^ in colonic epithelial cells). This would better address the importance of ACSS2 in KRAS^G12V^ mutant cells versus other mutations and whether it plays a detrimental role in tumorigenesis.

A fourth potential limitation of this study is that the results are based on experiments done in colon epithelial cells, so it is uncertain whether the KRAS G12V mutation will exhibit similar signaling properties in other tumor types. However, a recent association study of KRAS genotype and clinicopathological findings of early-resected NSCLC tumors suggested that the G12V genotype was closely associated with enhanced fatty acid and amino acid metabolism.^48^ Although not explicitly stated in this study, both pathways are consistent with elevated mTORC1 activity.

Finally, with respect to the clinical utility of our findings, it is possible that, as an adenoma acquires more mutations and progresses to carcinoma, the unique sensitivity of KRAS G12V cells to ACSS2 inhibition may diminish. p53 mutations are found in 50%–75% of CRC cases and occur at later stages of tumor progression.^49^ We do not know if differences in ACSS2 expression and acetate utilization between KRAS mutations are maintained when p53 mutations are introduced. P53 plays a regulatory role in lipogenesis and cholesterogenesis by transcriptionally inhibiting SREBP expression.^50–52^ Therefore, the loss of the p53 tumor suppressor may lead to increased SREBP and ACSS2 expression in all mutants. This might dilute the difference in ACSS2 expression between KRAS mutants and could potentially explain why it has not been reported previously in human CRC. However, G12V mutant cells may still maintain their enhanced ability to shift to acetate utilization when stressed, making them less responsive to chemotherapy, thus providing an opportunity to improve treatment by targeting acetate metabolism. Another possibility is that the loss of p53 may make other KRAS mutants more sensitive to ACSS2 inhibition. Whether p53 KO in KRAS-mutant cells elicits differences in ACSS2 expression and acetate utilization remains unexplored.

## RESOURCE AVAILABILITY

### Lead contact

Requests for further information and resources and reagents should be directed to and will be fulfilled by the lead contact, Dr. Jonathan Chernoff (J_Chernoff@fccc.edu).

### Materials availability

All unique reagents generated during this study are available from the lead contact without restriction.

### Data and code availability

- RNA-seq data have been deposited at GEO and are publicly available as of the date of publication. Proteomic data have been deposited at PRIDE and are publicly available as of the date of publication. Accession numbers are listed in the key resource table. http://www.ebi.ac.uk/pridereviewer_pxd050157@ebi.ac.uk https://www.ncbi.nlm.nih.gov/geo/info/update.html
- All data reported in this paper will be shared by the lead contact upon request.
- This paper does not report original code.
- Any additional information required to reanalyze the data reported in this paper is available from the lead contact upon request.

## STAR★METHODS

### EXPERIMENTAL MODEL AND STUDY PARTICIPANT DETAIL

#### Cell lines

Generation isogenic mouse colon epithelial cell lines are discussed in detail below. Cells were cultured in RPMI with 10% FBS, 1% Penicillin/Streptomycin. The LIM1215 and SW48 KRAS mutant isogenic cell lines were from Horizon Discovery. SW48 cells were cultured in RPMI supplemented with 10% FBS and 1% penicillin/streptomycin. LIM1215 cells were cultured in RPMI (with 2 mM L-Glutamine and 25 mM HEPES) supplemented with 10% FBS, 1% penicillin/streptomycin, 0.6 μg/mL insulin, 1 μg/mL hydrocortisone, and 10 μM 1-Thioglycerol. Sodium acetate or 250X Cholesterol was added where indicated. Cells were monitored for mycoplasma every 6 weeks using a Universal Mycoplasma Detection Kit. All experiments were performed on cells passaged under 20 times.

#### Animal studies

All animal studies were approved by the Fox Chase Cancer Center Institutional Animal Care and Use Committee (IACUC protocol number #22–14. Mice were randomly divided into the following arms based on the genetic background of the injected cell line: *Apc*^−/−^; *Kras*^*WT/G12D*^; *Acss2*^*WT*^, *Apc*^−/−^; *Kras*^*WT/G12V*^; *Acss2*^*WT*^, *Apc*^−/−^; *Kras*^*WT/G12D*^; *Acss2*^−/−^, *Apc*^−/−^; *Kras*^*WT/G12V*^; *Acss2*^−/−^. There were at least eight mice in each group (four males and four females). Tumors were measured using a caliper every three days for 28 days after reaching 150mm^3^. The tumor volume was calculated using the following formula: [(width)^2^ × (length)]/2. Student’s t-test was performed for statistical analysis of tumor weights at the end of the experiment (day 28), *p* values ≤0.05 were considered significant. All mice were maintained under defined-flora pathogen-free conditions at the AAALAC-approved Animal Facility of the Fox Chase Cancer Center, and monitored for signs of sickness, animal distress, or weight loss of more than 10%, per the local Institutional Animal Care and Use Committee (IACUC) guidelines.

### METHOD DETAIL*S*

#### Generation of isogenic mouse colon epithelial cell lines

An allelic series of *Kras* mutations was generated using a CRISPR-Cas knockin method (Figure S1).^22^ In short, stable C57BL/6 *Apc*^−/−^, *Kras*^*WT/G12D*^ cell line expressing Cas9 and BFP was established. This cell line was electroporated with 1 μg U6sgBFP-U6sg*Kras*^*G12D*^-pX333-HC-Red plasmid, 1 μL of 10 μM ssODN (*GFP*), and 1 μL of 10 μM ssODN (*Kras*^*G12V/G12R/G13D*^). Benchling CRISPR Design Tool (https://www.benchling.com) was used to design sgRNA and HDR templates for edits. Guides were selected based on their Off-target and On-target scores (0–100, higher is better). Off-target scores above 50, and On-target scores above 60 are considered to be good guides.^55,56^ ~72 h after transfection, GFP-positive cells were isolated, and single-cell sorted into a 96 well plate for clonal expansion. The remaining pulled GFP+ cells were analyzed by PCR and Sanger sequencing to verify desired *Kras*^*G12D*^ allele edit. Positive clones were expanded and further validated by PCR and Sanger sequencing. Candidate off-target sites were computationally identified using Cas-OFFinder (http://www.rgenome.net/cas-offinder) and IDT CRISPR genome editing tool (https://www.idtdna.com/page/products/crispr-genome-editing) for each sgRNA. Primary clones used in this study were evaluated for off-target effects by amplifying regions using PCR and Sanger sequencing the top 5 predicted sites for *BFP* sgRNA and top 7 for *Kras* sgRNA.

#### Flank implantation

5× 10^5^ *Apc*^−/−^; *Kras*^*WT/G12D*^ or *Apc*^−/−^; *Kras*^*WT/G12V*^ cells in 1:1 Matrigel: PBS were injected into the right flank of 6-week-old C57BL/6 mice. Once tumors reached approximately 100 mm^3^, mice were treated for 24 days. Trametinib was administered daily orally (P.O.) at 0.2 mg/kg dissolved in 0.5% hydroxypropylmethylcellulose, and 0.2% Tween-80. The ACSS2 inhibitor was administered every 2 days via I.P. at 15 mg/kg and dissolved in 5% DMSO, 40% PEG-300, 5% Tween-80, 50% H_2_O.

#### xCELLigence proliferation assay

Roughly 500 cells/well were seeded into a 16-well PET E-Plate in 100 μL of medium. Following seeding, the cells were monitored every hour for 120 h to measure their proliferation, attachment, and spreading. Doubling time was calculated using the RTCA software based on the logarithmic phase of the growth curve.

#### Active Ras detection

The Ras-GTP pulldown assay was performed according to the manufacturer’s instructions. Briefly, the cells were cultured overnight and harvested at ~80% confluency. Cells were washed with ice-cold PBS and harvested with a scraper in 0.5 mL Lysis/Binding/Wash Buffer plus 1 mM PMSF (per 10 cm plate) and collected into a microcentrifuge tube. The tubes were vortexed briefly and incubated on ice for 5 min. The tubes were microcentrifuged at 16,000 × *g* at 4°C for 15 min, and supernatant containing the cell lysate was transferred to a fresh tube. The protein concentration was measured by the BCA protein quantification assay. Glutathione resin was swirled thoroughly to resuspend the agarose beads, then 100 μL of the 50% resin slurry was added to the spin cup with collection tube. Tubes were spun down at 6,000 × g for 30 s. Beads were washed using 40 μL of 1X Lysis/Binding/Wash buffer and centrifuged at 6,000 × *g* for 30 s 80 μg of GST-Raf-RBD was added to the spin cup containing the glutathione resin, followed by the addition of 500 μg of total protein. The remaining total protein was used for input controls. The mixture was incubated at 4°C for 1 h with gentle rocking and then spun down at 6,000 × *g* for 30 s. Resin was washed 3 times by adding 400 μL of 1X Cell Lysis/Binding/Wash Buffer and centrifugation at 6,000 × *g* for 30 s 50 μL of reducing sample buffer (100 mM DTT in 2X SDS Sample Buffer) was added to the resin, vortexed, incubated for 2 min at room temperature, then spun down at 6,000 × *g* for 2 min. Finally, eluted samples were heated for 5 min at 95°C. 10 μL of the eluted sample was used for western blot analysis.

#### Western blotting

Cells were harvested at approximately 80% confluence using RIPA buffer with protease and phosphatase cocktail inhibitors. Cells were incubated on ice for 10 min, sonicated with 3 pulses for 10 s each at 20% intensity using a 550 sonic Dismembrator (Fisher Scientific), centrifuged for 15 min at 15,000 × *g* at 4°C, and supernatants were transferred to a fresh tube. Protein quantification was performed using the BCA protein assay and samples were diluted in sample buffer (1X Laemmli Buffer, 5% 2-mercaptoethanol). 20 μg of the lysate was loaded onto a 4–20% gradient Mini-PROTEAN TGX gel and transferred to a nitrocellulose membrane for 1 h. Membranes were blocked using blocking buffer (5% fat-free milk in TBST) for 1h at RT and then incubated with primary antibodies diluted 1:1000 in blocking buffer overnight at 4°C. The membranes were then incubated with secondary antibody at 1:10,000 dilution in blocking buffer and washed four times with TBST. Membranes were imaged using FluorChem E (ProteinSimple). Band intensity analysis was performed in ImageJ.

#### Cell viability assays

Roughly 1,000 mouse colon epithelial cells and 2,500 LIM1215 or SW48 cells were seeded into a white opaque 96-well plate. Next day, media was changed to medium supplemented with 2% FBS and inhibitor or DMSO. Readout of cell proliferation was adopted for cell growth properties, avoiding more than 80% confluence in the control wells. Cells were then cultured for 72–120 h, depending on their individual doubling times. The number of living cells was quantified by adding CellTiter-Glo, according to the instructions. The plates were read using an EnVision 2102 Multilabel Reader (PerkinElmer). EC50 values were calculated using GraphPad Prism 9 based on the inflection point of a curve. Synergy scores were determined using the Synergy Finder+ web application.^57^

#### RNA sequencing

Cells were harvested at ~80% confluence, then lysed in TRIzol reagent and chloroform according to the manufacturer’s instructions. Samples were DNase-treated and sequencing libraries were constructed using the TruSeq RNA Sample pre-kit-V2 and single-read sequenced (75 cycles) on HiSeq2500 (Illumina). Reads were aligned to the mouse mm10 genome using Tophat2.^58^ The cufflinks algorithm was implemented to assemble transcripts and estimate their abundance.^59^ Cuffdiff was used to statistically assess expression changes; genes with a false discovery rate of <0.05 and fold change of ≥2 were considered differentially expressed.^60^ Analysis of differentially expressed genes among genotypes was conducted with the SuperExactTest package in R.^61^

#### Total proteome analysis

Cells were harvested using lysis buffer (50 mM HEPES pH 8.0, 4% SDS). Protein quantification was performed using the BCA protein assay and 100 μg of protein was digested using LysC for 3 h, followed by trypsin overnight. The following day, digested peptides were isolated using C-18 and PGC columns, dried and washed with ethyl acetate. 3 μg was then resuspended in 0.1% formic acid and separated using a Thermo Scientific RSLC nano Ultimate 3000 LC on a Thermo Scientific Easy-Spray C-18 PepMap 75 mm × 50 cm C-18 2 mm column. A 305 min gradient of 2–20% (180 min) 20%–28% (45 min) 28%–48% (20 min) acetonitrile with 0.1% formic acid was run at 300 nL/min at 50C. Eluted peptides were analyzed using Thermo Scientific Q Exactive or Q Exactive plus mass spectrometers utilizing a top 15 methodology in which the 15 most intense peptide precursor ions were subjected to fragmentation. The AGC for MS1 was set to 3×10^6^ with a maximum injection time of 120 ms, the AGC for MS2 ions was set to 1×10^5^ with a maximum injection time of 150 ms, and the dynamic exclusion was set to 90 s. Raw data analysis of LFQ experiments was performed by the Fox Chase Cancer Center Biostatistics and Bioinformatics Facility.

#### qRT-PCR

TRIzol reagent solution and chloroform were used to isolate RNA from cells according to manufacturer’s instructions. qRT-PCR on diluted cDNA was performed with inventoried TaqMan Gene Expression Assays on the QuantStudio 7 Pro System. TaqMan Gene Expression Assay probes were used to assess changes in gene expression, including *Acss2*, *Hmgcs1*, *Srebf1*, and *Rplp0* (control). The samples were run in triplicates. The Student’s *t*-tests was used for statistical analyses.

#### Intracellular cholesterol quantification

Intracellular cholesterol quantification was performed using the Cholesterol/Cholesterol Ester-Glo assay according to the manufacturer’s instructions. Briefly, cells were plated in two white opaque 96-well plate and cultured until ~80% confluency. One of the plates was used for normalization, the other was used for cholesterol quantification. For normalization, cell viability was determined using CellTiter-Glo as described above. To measure cholesterol, the medium was removed, cells were washed twice with PBS, and 50 μL of Cholesterol Lysis Solution was added to each well followed by incubation for 30 min at 37°C. Then, 50 μL of Cholesterol Detection Reagent with or without esterase was added to each well, plate was shaken for 30–60 s at a low rpm and incubated for 1 h at RT. Plates were read using EnVision 2102 Multilabel Reader (PerkinElmer).

#### Cellular fractionation

Cellular fractionation was performed using the NE-PER Nuclear and Cytoplasmic Extraction Kit according to the manufacturer’s instructions. Briefly, the cells were grown to approximately 80% confluence, harvested using TrypLE Express Enzyme, and centrifuged at 500 × *g* for 5 min. After removing the supernatant, the pellet was resuspended in ice-cold CER I buffer, vortexed, and then incubated on ice for 10 min. Ice-cold CER II was added to the mixture, vortexed, incubated on ice for 1 min, and centrifuged at 16,000 × *g* for 5 min. The supernatant (cytoplasmic fraction) was transferred to a new tube. The remaining pellet was resuspended in ice-cold NER buffer and incubated on ice for 40 min with intermittent vortexing every 10 min. Afterward, samples were centrifuged at the 16,000 × *g* for 10 min. Nuclear extract was transferred to a new tube. Protein quantification was performed using the BCA protein assay and samples were diluted to similar concentrations in sample buffer (1X Laemmli Buffer, 5% 2-mercaptoethanol). 20 μg of lysate was loaded onto a 4–20% gradient Mini-PROTEAN TGX gel and western blotting was performed as described previously.

#### Glucose and acetate labeling

[U-^13^C]glucose and [1,2-^13^C]acetate incorporation into acetyl-CoAs was analyzed in cells incubated in DMEM without glucose, glutamine, or pyruvate with 10% dialyzed FBS in the presence of 5 mM [U-^13^C]glucose and 1 mM unlabeled acetate or 5 mM unlabeled glucose and 5 mM [1,2-^13^C]acetate for 24 h. The next day, the medium was aspirated and 1 mL of ice-cold 10% trichloroacetic acid (w/v) in water was added. Cells were scraped into a new tube and frozen at −80°C. For relative acetyl-CoA determination, cells were incubated under the same conditions in the absence of labeled substrate. The cell volume and concentration were determined using a TC20 Automated Cell Counter.

#### Acetyl-CoA measurements

The short-chain acyl-CoA internal standard was generated in yeast as previously described.^62^ Acyl-CoAs were analyzed using liquid chromatography-high-resolution mass spectrometry (LC-HRMS) as previously described.^63^ 50 μL of short-chain acyl-CoA ISTD was added and the cell suspensions were sonicated with 5 × 0.5-s pulses at 50% intensity (Fisherbrand Sonic Dismembrator Model 120 with Qsonica CL-18 sonicator probe). Lysates were centrifuged at 17000 × *g* for 10 min at 4°C and clarified lysates were transferred to a deep-well 96-well plate for loading in a Tomtec Quadra4 liquid handling workstation. On the liquid handling workstation, lysates were applied to an Oasis HLB 96-well elution plate (Waters) (30 mg of sorbent per well) pre-conditioned and equilibrated with 1 mL methanol and 1 mL water, respectively. After de-salting with 1 mL of water, acetyl-CoA was eluted into a deep-well 96-well plate using 1 mL of 25 mM ammonium acetate in methanol. The eluent was evaporated and dried under nitrogen gas. The dried LC-HRMS samples were resuspended in 50 μL 5% (w/v) sulfosalicylic acid in water. 5 μL injections of each sample were analyzed via LC-HRMS, using an Ultimate 3000 quaternary ultra-high performance liquid chromatograph coupled with a Q Exactive Plus mass spectrometer (Thermo Scientific) as previously described.^64^ A modified gradient using solvent A (5 mM ammonium acetate in water), solvent B (5 mM ammonium acetate in 95:5 (v/v) acetonitrile: water) and solvent C (0.1% (v/v) formic acid in 80:20 (v/v) acetonitrile: water). Data were acquired using XCalibur 4.0, analyzed using Tracefinder 5.1, and corrected for normal isotopic distribution using FluxFix.^65^

#### *Acss2* CRISPR-mediated knockout

Approximately 100,000 cells (80% confluency) were used per electroporation reaction. On the day of electroporation, the cells were washed twice with PBS and detached using TrypLE Express. Ribonucleoprotein (RNP) complexes were assembled in a ratio of 6:1 (sgRNA:Cas9) in 7 μL for a single reaction. The RNP solution was combined with 5 μL of cell suspension containing 100,000 cells and electroporated in a 10 μL tip using Neon Transfection System (Thermo). Immediately thereafter, electroporated cells were transferred to pre-warmed wells of a 6 well plate and cultured for 2–3 days. Confirmation of *Acss2* knock-out was performed by western blotting 72 h after electroporation. Clones with successful knockouts were retained for subsequent analysis.

### QUANTIFICATION AND STATISTICAL ANALYSIS

GraphPad Prism 9 was used for plotting and statistical analysis. Western Blot band intensity analysis was performed in ImageJ. Synergy scores were determined using the Synergy Finder+ web application. The number of replicates (n) and the statistical test used for each experiment are indicated in the figure legend. *p values of <0.05 are considered statistically significant.

## Supplementary Material

1

## Figures and Tables

**KEY RESOURCES TABLE:** 

REAGENT or RESOURCE	SOURCE	IDENTIFIER

Antibodies		

Rabbit polyclonal anti AKT	Cell Signaling Technology	Cat# 9272; RRID:AB_329827; 1:1000
Rabbit anti phospho-AKT (S473)	Cell Signaling Technology	Cat# 4060; RRID:AB_2315049; 1:2000
Rabbit anti phospho-AKT (T308)	Cell Signaling Technology	Cat# 13038; RRID:AB_2629447; 1:1000
Rabbit anti RAS	Cell Signaling Technology	Cat# 67648; RRID:AB_2910195; 1:1000
Rabbit anti AceCS1	Cell Signaling Technology	Cat# 3658; RRID:AB_2222710; 1:1000
Mouse anti ACLY	Cell Signaling Technology	Cat# 13390; RRID:AB_2798203; 1:1000
Rabbit polyclonal anti phospho-ACLY (S455)	Cell Signaling Technology	Cat# 4331; RRID:AB_2257987; 1:1000
Mouse anti SREBP	Santa Cruz Biotechnology	Cat #sc-13551; RRID:AB_628282; 1:1000
Rabbit anti RAPTOR	Cell Signaling Technology	Cat# 2280; RRID:AB_561245; 1:1000
Rabbit anti ERK1/2	Cell Signaling Technology	Cat# 4695; RRID:AB_390779; 1:2500
Rabbit anti phospho-ERK1/2 (T202/Y204)	Cell Signaling Technology	Cat# 9101; RRID:AB_331646; 1:2500
Rabbit anti ACC	Cell Signaling Technology	Cat# 4190; RRID:AB_10547752; 1:1000
Rabbit anti phospho-ACC (S79)	Cell Signaling Technology	Cat# 11818; RRID:AB_2687505; 1:1000
Rabbit anti Ras-G12D	Cell Signaling Technology	Cat# 14429; RRID:AB_2728748; 1:1000
Rabbit anti Ras-G12V	Cell Signaling Technology	Cat# 14412; RRID:AB_2714031; 1:1000
Rabbit anti mTor	Cell Signaling Technology	Cat#2983; RRID:AB_2105622; 1:1000
Rabbit anti phospho-mTor (S2448)	Cell Signaling Technology	Cat#2971; RRID:AB_330970; 1:1000
Rabbit anti S6	Cell Signaling Technology	Cat#2217; RRID:AB_331355; 1:1000
Rabbit anti phospho-S6 (S235/236)	Cell Signaling Technology	Cat#4857; RRID:AB_2181035; 1:1000
Rabbit anti 4E-BP1	Cell Signaling Technology	Cat#9452; RRID:AB_331692; 1:1000
Rabbit anti phospho-4E-BP1 (S65)	Cell Signaling Technology	Cat#9451; RRID:AB_330947; 1:1000
Rabbit anti-acetylated lysine	Invitrogen	Cat# MA5-33031; RRID:AB_2810124; 1:1000
Rabbit polyclonal anti Lamin A/C	Cell Signaling Technology	Cat #2032; RRID:AB_2136278; 1:1000
Rabbit anti GAPDH	Cell Signaling Technology	Cat# 2118; RRID:AB_561053; 1:2500
Peroxidase AffiniPure Goat Anti-Mouse IgG (H + L)	Jackson Immunoresearch	Cat# 115-035-003; RRID:AB_10015289; 1:10000
Peroxidase AffiniPure Goat Anti-Rabbit IgG (H + L)	Jackson Immunoresearch	Cat# 115-036-003; RRID:AB_2338518; 1:10000

Bacterial and virus strains		

Stellar Competent Cells	Takara	Cat# 636763

Chemicals, peptides, and recombinant proteins		

DMSO	Fisher	Cat# BP231-100
Acetate	ThermoScientific	Cat# 537462
Glucose	Sigma	Cat# G7021
[U-^13^C]glucose	Cambridge Isotope Laboratories	Cat# CLM-1396-1
[1,2-^13^C]acetate	Cambridge Isotope Laboratories	Cat# CLM-440-1
Trichloroacetic acid (w/v)	Sigma	Cat# T6399
DMEM Medium	Gibco	Cat# 10566-016
RPMI Medium	Gibco	Cat# 72400-047
Fetal Bovine Serum	HyClone	Cat# SH30071.03
Penicillin Streptomycin	Gibco	Cat# 15150-122
ACSS2 inhibitor	Selleck	Cat# S8588
Acetyl-CoA, lithium salt	Sigma-Aldrich	Cat# A2181
5-sulfosalicylic acid	Sigma-Aldrich	Cat# A2130
LC/MS grade acetonitrile	Fisher Scientific	Cat# A998-1
Formic acid	Fisher Scientific	Cat# A117-05AMP
Methanol	Fisher Scientific	Cat# 4581
TRI reagent solution	Invitrogen	Cat# AM9738
Chloroform	Sigma-Aldrich	Cat# 650498
TrypLE Express Enzyme	Gibco	Cat# 12605010
250X Cholesterol	Gibco	Cat# 12531-018
Protease inhibitor cocktail	Sigma-Aldrich	Cat# 11836170001
Phosphatase inhibitor cocktail	Sigma-Aldrich	Cat# 78420
Lipofectamine^™^ 3000	Invitrogen	Cat# L3000001
SpCas9 nuclease	Synthego	Cat SpCas9
L-glutamine	Gibco	Cat# 25030081
HEPES	Gibco	Cat# 15630130
Insulin	Lonza	Cat# BE02-033E20
Hydrocortisone	Stemcell	Cat# 74142
Thioglycerol	Sigma Aldrich	Cat# M6145
PMSF	Thermo Scientific	Cat# 36978
Dithiothreitol (DTT)	Thermo Scientific	Cat# R0861
RIPA Buffer	Sigma Aldrich	Cat# R0278
4X Laemmli Buffer	BioRad	Cat# 1610747
2-mercaptoethanol	Fisher Scientific	Cat# 21985023
Non-fat milk	BioRad	Cat# 1706404
Lys-C Mass Spec Grade	Promega	Cat# VA117A
Acetonitrile	Thermo Scientific Chemicals	Cat# 047138.M1
dialyzed FBS	Gibco	Cat# A3382001
Ammonium acetate	Sigma	Cat# 533004
Lipofectamine 3000	Invitrogen	Cat# L3000015
Puromycin	Gibco	Cat# A1113803
Polybrene	Sigma-Aldrich	Cat# TR-1003
Trametinib	Selleck	Cat# S2673
Hydroxypropyl methylcellulose	Thermo Scientific Chemicals	Cat# 044779.A1
Tween-80	Sigma Aldrich	Cat# P1754
Polyethylene glycol 300 (PEG-300)	Selleck	Cat# S6704

Critical commercial assays		

Mycoplasma Detection Kit	ATCC	Cat# 30-1012K
TruSeq RNA Sample pre-kit-V2	Illumina	Cat# RS-122-2001
Cholesterol/Cholesterol Ester-Glo assay	Promega	Cat# J3190
Active Ras Detection Kit	Cell Signaling Technology	Cat# 8821
Pierce BCA Protein Assay Kit	Thermo Scientific	Cat# 23225
CellTiter-Glo 2.0	Promega	Cat# G9241
NE-PER Nuclear and Cytoplasmic Extraction Kit	Thermo Scientific	Cat# 78833

Deposited data		

KRAS Transcriptomics	This study	Geo: GSE261333
KRAS Proteomics	This study	Pride: PXD050157

Experimental models: Cell lines		

HEK293	ATCC	Cat# CRL-1573;
LIM1215 KRAS^WT/WT^	Sigma Aldrich	Cat# 10092301
LIM1215 Kras^WT/G12D^	Horizon Discovery	Cat# HD 116-005
LIM1215 Kras^WT/G12V^	Horizon Discovery	Cat# HD 116-006
LIM1215 Kras^WT/G13D^	Horizon Discovery	Cat# HD 116-009
SW48 Kras^WT/WT^	ATCC	Cat# CCL231
SW48 Kras^WT/G12D^	Horizon Discovery	Cat# HD 103-011
SW48 Kras^WT/G12V^	Horizon Discovery	Cat# HD 103-007
CRC adenoma cells *Apc^−/−^::Kras^WT/G12D^*	K. Haigis	NA
CRC adenoma cells *Apc^−/−^::Kras^WT/G12V^*	Budagyan and Chernoff^22^	NA
CRC adenoma cells *Apc^−/−^::Kras^WT/G12R^*	Budagyan and Chernoff^22^	NA
CRC adenoma cells *Apc^−/−^::Kras^WT/G13D^*	Budagyan and Chernoff^22^	NA

Experimental models: Organisms/strains		

C57BL/6J	Jackson Laboratories	Cat# JAX:00664

Oligonucleotides		

*Acss2* sgRNA	Synthego	NA
*Kras* forward primer 5′-GGGTAGGTGTTGGGATAGCTGTCGACAAGC-3′	Integrated DNA Technologies	NA
*Kras* reverse primer 5′-CTTTACAAGCGCACGCAGACTGTAGAGC-3′	Integrated DNA Technologies	NA
*Kras* sgRNA top primer 5′- CACCGTGGTTGGAGCTGATGGCGT-3′	Integrated DNA Technologies	NA
*Kras* sgRNA bottom primer 5′-AAACACGCCATCAGCTCCAACCAC-3′	Integrated DNA Technologies	NA
BFP sgRNA top primer 5′- CACCGCACTGCACCCCGTGGCTCA-3′	Integrated DNA Technologies	NA
BFP sgRNA bottom primer 5′-AAACTGAGCCACGGGGTGCAGTGC-3′	Integrated DNA Technologies	NA
ssODN *GFP* 5′CACCACCGGCAAGCT GCCCGTGCCCTGGCCCACCCTCGTGACCACCCTGACCTACGGCGTGCAGTGCTTCAGCCGCTACCCCGACCACATGAAGCAGCAC -3′	Integrated DNA Technologies	NA
ssODN *Kras*^*G12V*^ 5′TGCTGAAAATGACTGAGTATAAGCTTGTGGTGGTTGGCGCCGTTGGAGTAGGCAAGAGCGCCTTGACGATACAGCTAATTCA-3′	Integrated DNA Technologies	NA
ssODN *Kras*^*G12R*^ 5 TGCTGAAAATGACTGAGTATAAGCTTGTGGTGGTTGGCGCCGTGTGAGTAGGCAAGAGCGCCTTGACGATACAGCTAATTCA-3′	Integrated DNA Technologies	NA
ssODN *Kras*^*G13D*^ 5 TGCTGAAAATGACTGAGTATAAGCTTGTGGTGGTTGGCGCCGGTGATGTAGGCAAGAGCGCCTTGACGATACAGCTAATTCA-3′	Integrated DNA Technologies	NA
*Kras* sequencing primer 5′TCTTGTGTGAGACATG-3′	Integrated DNA Technologies	NA
*Acss2* TaqMan Gene Expression Assay	Invitrogen	Cat# Mm00480101_m1
*Hmgcs1* TaqMan Gene Expression Assay	Invitrogen	Cat# Mm1304569_m1
*Srebf1* TaqMan Gene Expression Assay	Invitrogen	Cat# Mm00550338_m1
*Rplp0* TaqMan Gene Expression Assay	Invitrogen	Cat# Mm00725448_s1

Recombinant DNA		

pLentiCas9-P2A-BFP	Addgene	RRID:Addgene_149447
U6:sgBFP-U6:sg*Kras^G12D^*-pX333-HC-Red	Addgene	RRID:Addgene_149448
pLJMI-ACSS2	J.C. Alwine	Vysochan et al.^53^
pTRIPZ	Addgene	RRID:Addgene_206981
pTRIPZ shACSS2.1	K. Rathmell	Bacigalupa et al.^54^
pTRIPZ shACSS2.2	K. Rathmell	Bacigalupa et al.^54^

Software and algorithms		

GraphPad Prism	GraphPad Software, Inc.	www.graphpad.com/
Adobe Illustrator	Adobe Creative Cloud	https://www.adobe.com/creativecloud.html
Proteome Discoverer 2.2	Thermo Fisher Scientific	https://www.thermofisher.com/order/catalog/product/OPTION-30795
R Framework	Team RCR: A Language and Environment for Statistical Computing	http://www.R-project.org/
Python 3.7.3	Python Software Foundation	https://www.python.org/
Bio Render App	Bio Render	https://www.biorender.com/
XCalibur 4.0	Thermo Scientific	OPTON-30965
Tracefinder 5.1	Thermo Scientific	OPTON-31001
Cas-OFFinder	CRISPR RGEN Tools	http://www.rgenome.net/cas-offinder
IDT CRISPR	Integrated DNA Technologies	https://www.idtdna.com/page/products/crispr-genome-editing
Xcelligence RTCA software	Agilent	www.agilent.com
Synergy Finder+ web application	SynergyFinder	www.synergyfinder.org
Tophat2	Johns Hopkins University/Github	https://github.com/infphilo/tophat
Cufflinks	Github	https://github.com/cole-trapnell-lab/cufflinks

## Highlights

- KRAS G12V activates distinct signaling pathways in *Apc*-null mouse colon adenoma cells
- Cells expressing KRAS G12V exhibit decreased sensitivity to downstream pathway inhibitors
- KRAS G12V cells show enhanced dependance on ACSS2 to generate acetyl-CoA from acetate
- Targeting ACSS2 in KRAS G12V cells impacts tumorigenicity and sensitivity to MEK inhibition
