# Supplementary material for: KRAS G12V mutation-selective requirement for ACSS2 in colorectal adenoma formation
Source: Cell Rep. Author manuscript; Available in PMC 2025 May 20. (PMC12091147; doi:10.1016/j.celrep.2025.115444)
Supplement: 1 [file NIHMS2076697-supplement-1.pdf]

Cell Reports, Volume *44*

Supplemental information

KRAS G12V mutation-selective requirement for ACSS2 in colorectal adenoma formation

Konstantin Budagyan, Alexa C. Cannon, Adam Chatoff, Dorothy Benton, Alison M. Kurimchak, Daniela Araiza-Olivera, Anastasiia Gerasimova, Nathaniel W. Snyder, James S. Duncan, Cristina Uribe-Alvarez, and Jonathan Chernoff

Supplementary Figures

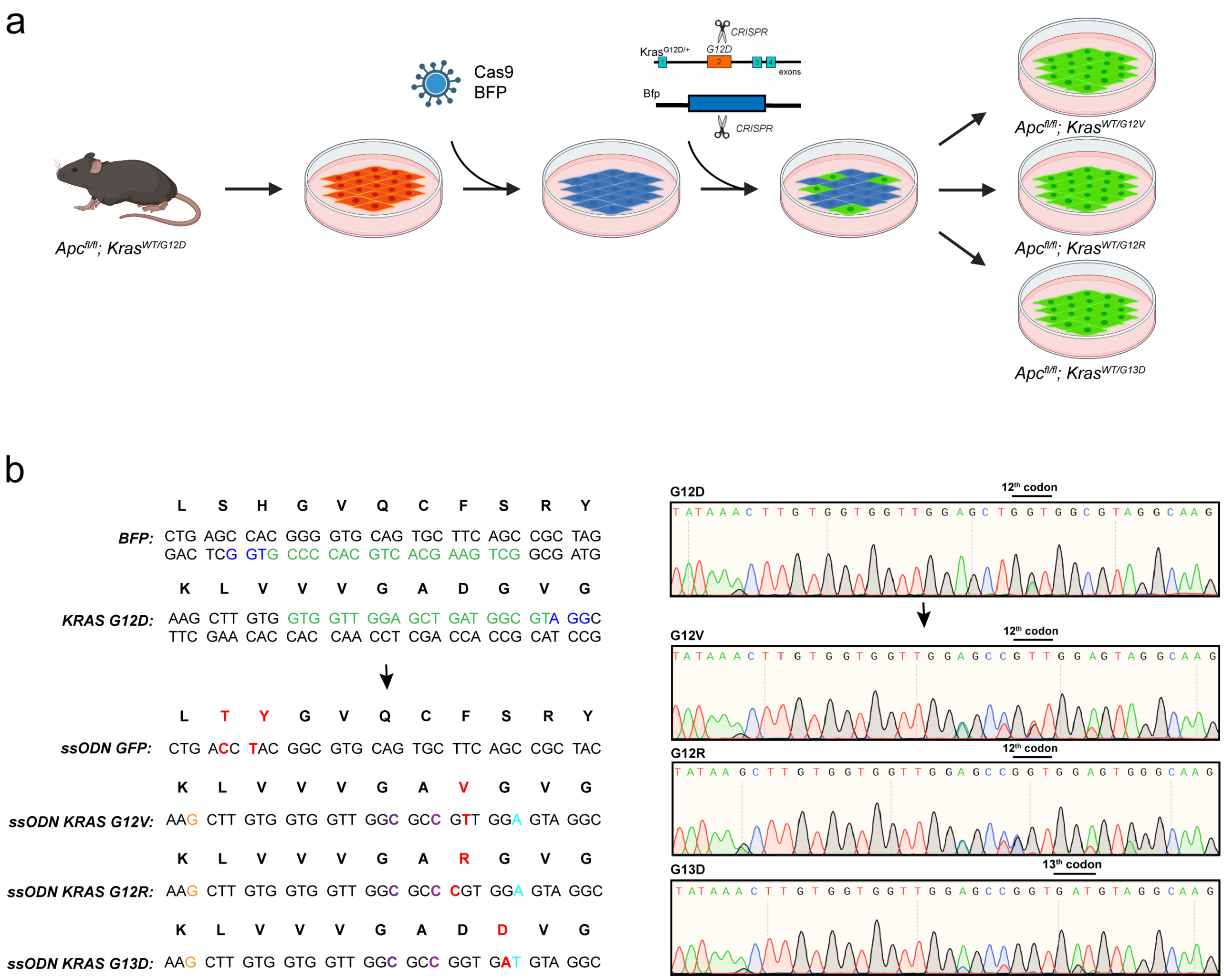

Supplementary Figure 1. Generation of the mouse isogenic cell line model.
A. Schematic of a fluorescent co-selection for CRISPR-driven *Kras* point editing. Mouse colon epithelial cells are first transduced with a lentivirus containing *Cas9* linked to *BFP*. BFP-positive cells are then transfected with a plasmid containing two sgRNAs targeting endogenous *BFP* and *Kras G12D* allele (green), along with two ssODN designed as templates for HDR.
B. Sequences of single cell clones containing desired edits.

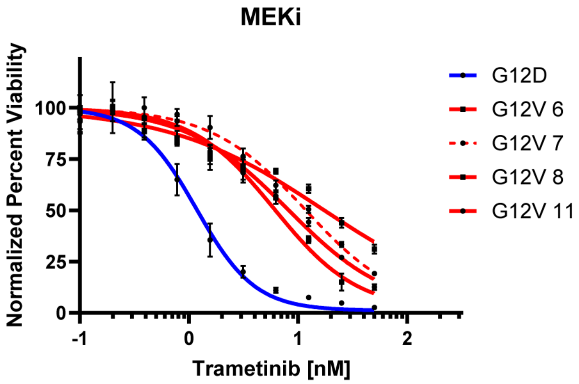

Supplementary Figure 2. Sensitivity of multiple KRAS G12V clones to trametinib. KRAS G12V Clone #7 was used for majority of the experiments in this study.

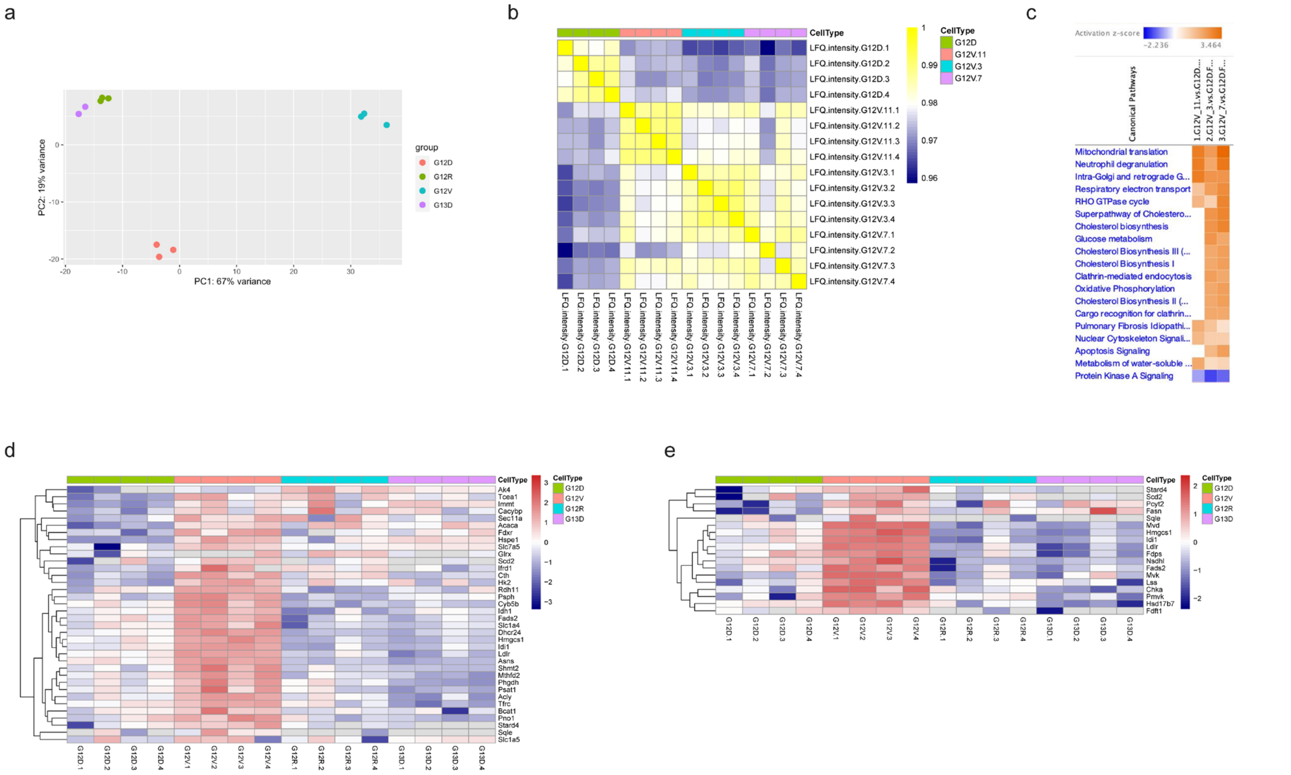

Supplementary Figure 3. Proteome analysis of KRAS mutant mouse colon epithelial cell clones.
A. Principal component analysis of genes with high variance in expression level, as determined by RNA sequencing.
B. Correlation matrix based on proteome analysis of 3 different KRAS G12V clones compared to KRAS G12D starting cell line.
C. Detailed heatmap of significantly enriched canonical pathways in KRAS G12V clones relative to KRAS G12D. FDR < 0.05, logFC > 1.5.
D-E. Detailed heatmap of significantly enriched hallmark gene sets for mTORC (**D**) and cholesterol homeostasis pathways (**E**) in KRAS G12D, G12R, or G13D cells relative to KRAS G12V, as determined by global proteomic analysis of KRAS mutant cells. FDR < 0.05, logFC > 2.

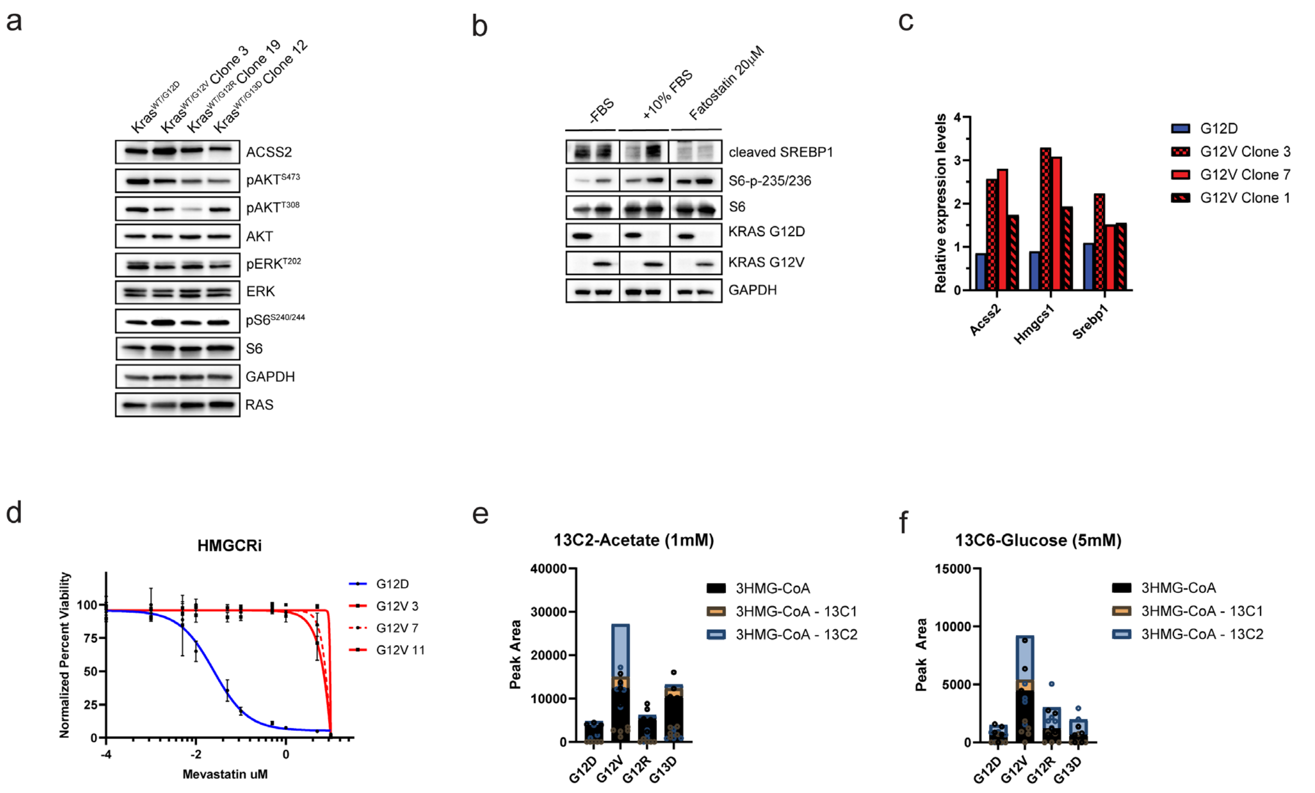

Supplementary Figure 4. KRAS G12V mouse colon epithelial cells show increased ACSS2 utilization to generate cholesterol.
A. Representative western blot for expression of lipogenic enzymes in KRAS mutant mouse colon epithelial cells using different clones cultured in normal serum media. Results are representative of three similar experiments
B. Representative western blot for expression of cleaved SREBP1 in KRAS G12D and KRAS G12V mouse colon epithelial cells in serum depleted and normal serum conditions, along with fatostatin, SCAP inhibitor that prevents SREBP1 cleavage.
C. qPCR of *Acss2*, *Hmgcs1*, *Srebp1* in three KRAS G12V clones compared to starting KRAS G12D cell line.
D. Sensitivity of KRAS G12D and G12V clones to mevastatin. Data shows Cell-Titer Glo luminescence averages, curves were fit with nonlinear regression in GraphPad Prism 9.
E-F. Stacked peaked area of labeled HMG-CoA using 13C2 acetate (**E**), or 13C6 glucose (**F**)

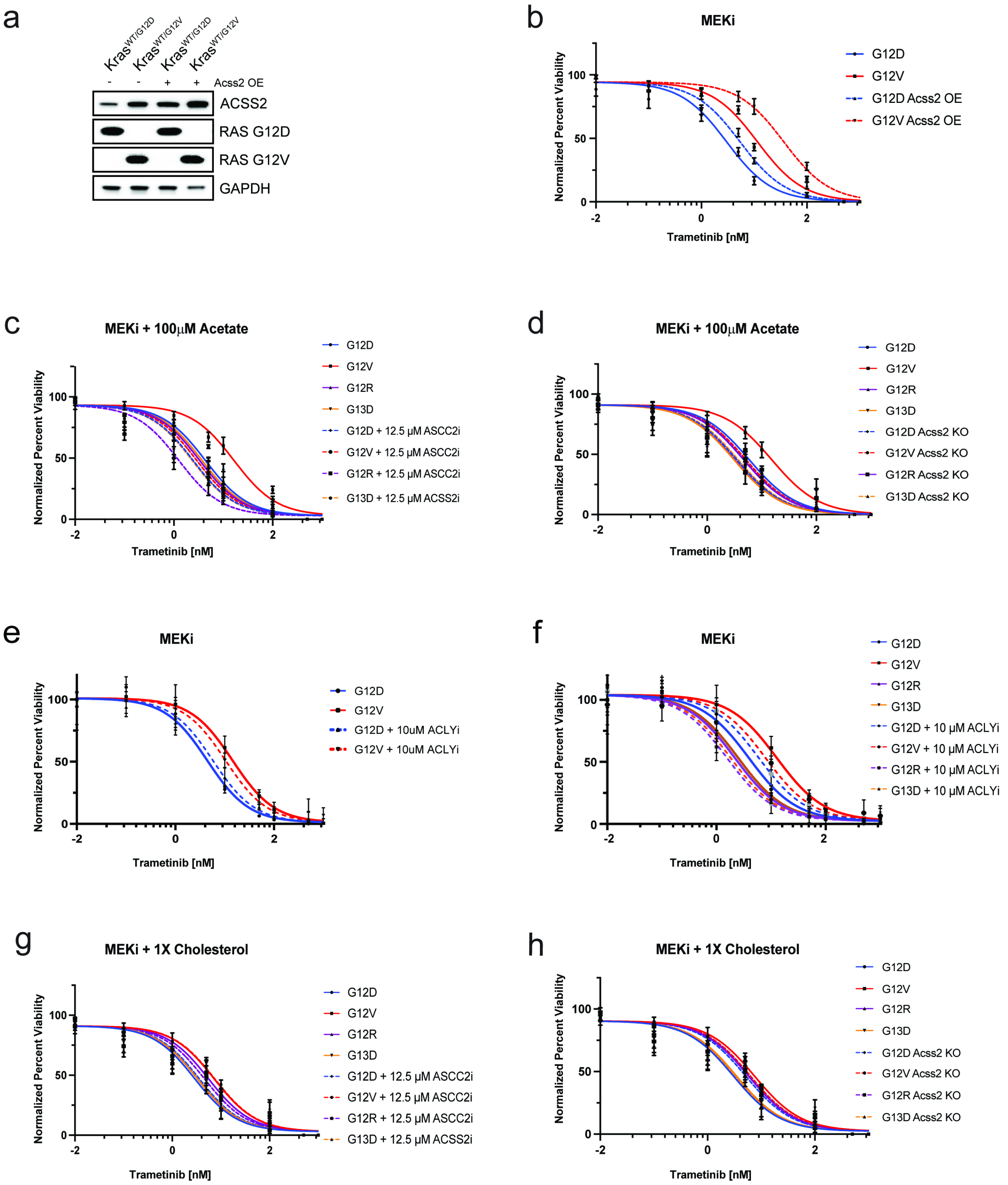

Supplementary Figure 5: ACSS2 decreases the sensitivity of KRAS G12V cells to MEK inhibition.
A. Representative western blot of ACSS2 overexpression in KRAS G12D and G12V cells
B. Sensitivity of KRAS mutant mouse colon epithelial cells with ACSS2 overexpression to MEK inhibition. Data shows Cell-Titer Glo luminescence averages, curves were fit with nonlinear regression in GraphPad Prism 9
C-D. Sensitivity of KRAS mutant mouse colon epithelial cells to MEK inhibition in combination with ACSS2 inhibitor cultured in media with 100μM acetate. Data shows Cell-Titer Glo luminescence averages, curves were fit with nonlinear regression in GraphPad Prism 9.
E-F. Sensitivity of KRAS mutant mouse colon epithelial cells to MEK inhibition in combination with ACLY inhibitor. Data shows Cell-Titer Glo luminescence averages, curves were fit with nonlinear regression in GraphPad Prism 9.
G-H. Sensitivity of KRAS mutant mouse colon epithelial cells to MEK inhibition in combination with ACSS2 inhibitor cultured in media with exogenous cholesterol. Data shows Cell-Titer Glo luminescence averages, curves were fit with nonlinear regression in GraphPad Prism 9.

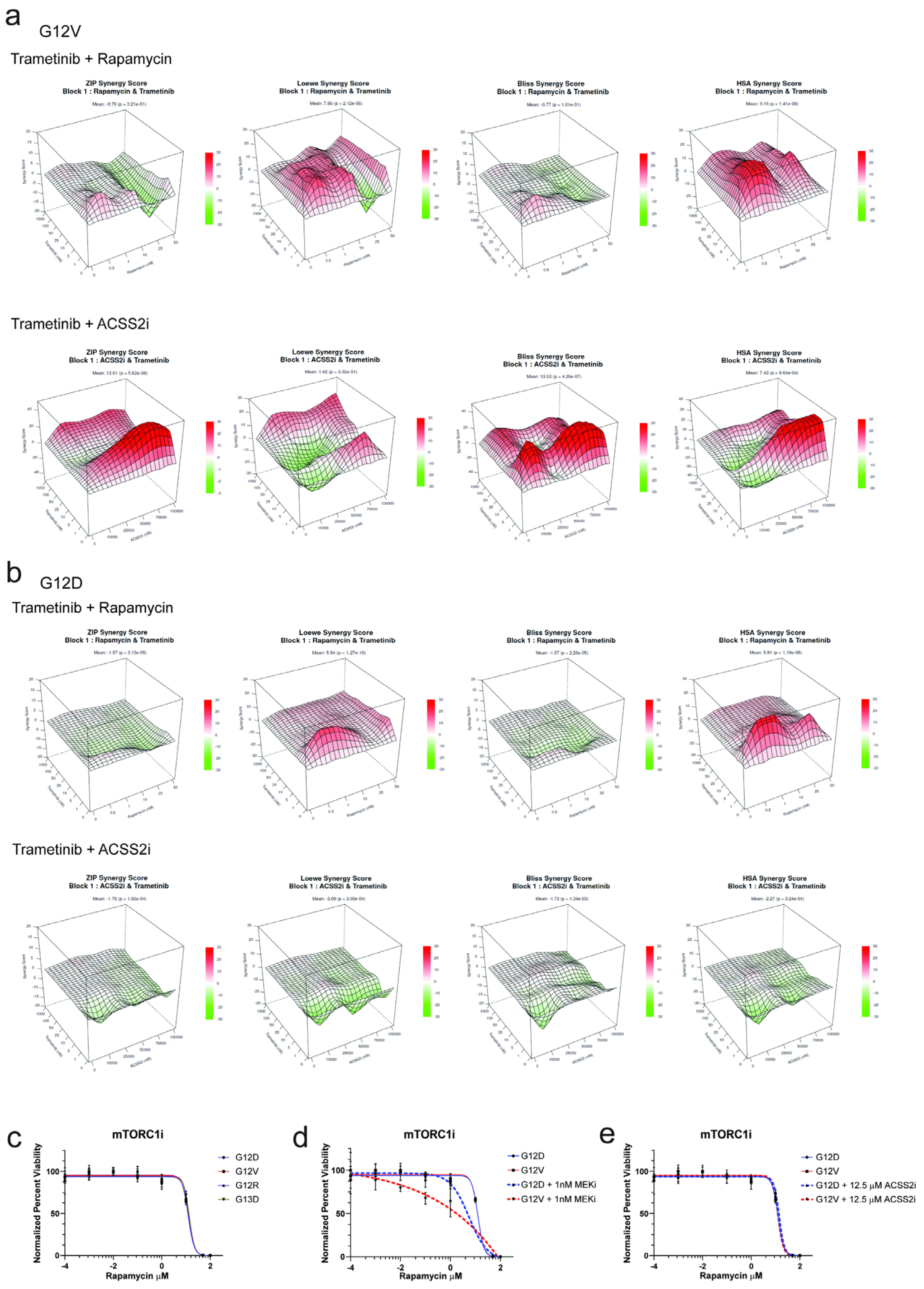

Supplementary Figure 6: ACSS2 inhibition synergizes KRAS G12V mouse colon epithelial cells to MEK inhibition.

A-B. Synergistic effect of MEK inhibition with either ACSS2 or mTOR inhibitors in KRAS G12D (**A**) and KRAS G12V(**B**) cells

C. Sensitivity of KRAS mutant mouse colon epithelial cells to mTORC1 inhibition. Data shows Cell-Titer Glo luminescence averages, curves were fit with nonlinear regression in GraphPad Prism 9.

D. Sensitivity of KRAS mutant mouse colon epithelial cells to mTORC1 inhibition in combination with MEK inhibitor. Data shows Cell-Titer Glo luminescence averages, curves were fit with nonlinear regression in GraphPad Prism 9.

E. Sensitivity of KRAS mutant mouse colon epithelial cells to mTORC1 inhibition in combination with ACSS2 inhibitor. Data shows Cell-Titer Glo luminescence averages, curves were fit with nonlinear regression in GraphPad Prism 9.

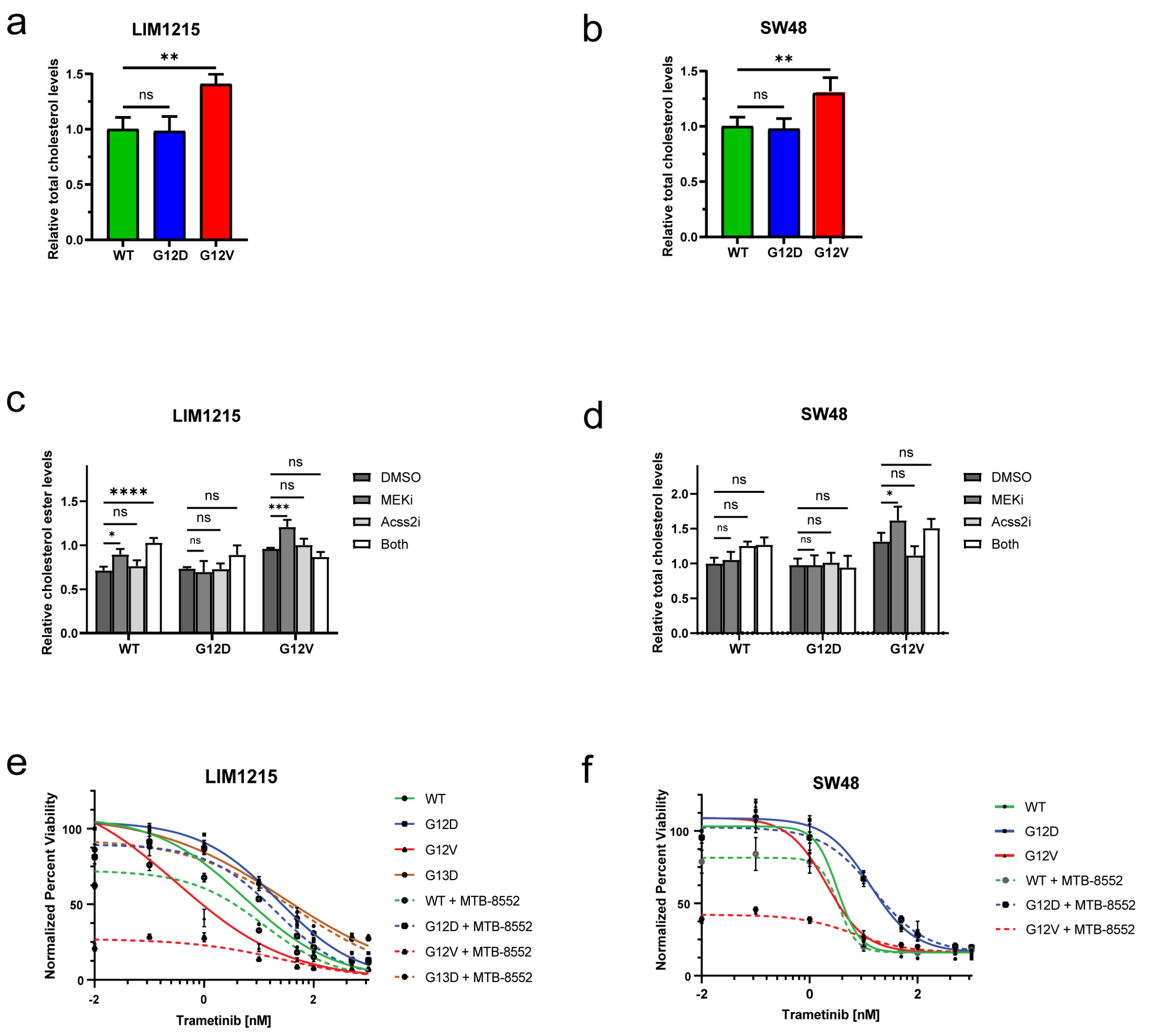

Supplementary Figure 7: Inhibition of ACSS2 sensitizes KRAS G12V human CRC cells to MEK inhibition.
A-B. Intracellular cholesterol ester levels in KRAS mutant human isogenic cell lines. Data shows Cholesterol Ester-Glo luminescence averages from three biological replicates. One way ANOVA, ns not significant, * p<0.05, *** p<0.0001.
C-D. Intracellular cholesterol ester levels in human isogenic KRAS mutant cell lines in response to MEK inhibition, ACSS2 inhibition, or the combination of the two. Data shows Cholesterol Ester-Glo luminescence averages from three biological replicates. One way ANOVA, ns not significant, * p<0.05, *** p<0.0001.
E-F. Sensitivity of KRAS mutant human isogenic cell lines to MEK inhibition in combination with ACSS2 inhibitor. Data shows Cell-Titer Glo luminescence averages, curves were fit with nonlinear regression in GraphPad Prism 9.
